# Investigations on SARS-CoV-2 Susceptibility of Domestic and Wild Animals Using Primary Cell Culture Models Derived from the Upper and Lower Respiratory Tract

**DOI:** 10.3390/v14040828

**Published:** 2022-04-16

**Authors:** Iris Färber, Johannes Krüger, Cheila Rocha, Federico Armando, Maren von Köckritz-Blickwede, Stefan Pöhlmann, Armin Braun, Wolfgang Baumgärtner, Sandra Runft, Nadine Krüger

**Affiliations:** 1 Department of Pathology, University of Veterinary Medicine, Foundation, 30559 Hannover, Germany; iris.verena.faerber@tiho-hannover.de (I.F.); johannes.krueger@tiho-hannover.de (J.K.); federico.armando@tiho-hannover.de (F.A.); sandra.runft@tiho-hannover.de (S.R.); 2 Infection Biology Unit, German Primate Center, Leibniz Institute for Primate Research, 37077 Göttingen, Germany; crocha@dpz.eu (C.R.); spoehlmann@dpz.eu (S.P.); nkrueger@dpz.eu (N.K.); 3 Department of Biochemistry, University of Veterinary Medicine Hannover, Foundation, 30559 Hannover, Germany; maren.von.koeckritz-blickwede@tiho-hannover.de; 4 Research Center for Emerging Infections and Zoonoses, University of Veterinary Medicine, Foundation, 30559 Hannover, Germany; 5 Faculty of Biology and Psychology, Georg-August-University, 37073 Göttingen, Germany; 6 Fraunhofer Institute for Toxicology and Experimental Medicine ITEM, 30625 Hannover, Germany; braun.armin@mh-hannover.de

**Keywords:** SARS-CoV-2, respiratory tract, animals, zoonosis, primary cell cultures, ACE2, TMPRSS2, CTSL, air–liquid interface, tissue explants

## Abstract

Several animal species are susceptible to SARS-CoV-2 infection, as documented by case reports and serological and in vivo infection studies. However, the susceptibility of many animal species remains unknown. Furthermore, the expression patterns of SARS-CoV-2 entry factors, such as the receptor angiotensin-converting enzyme 2 (ACE2), as well as transmembrane protease serine subtype 2 (TMPRSS2) and cathepsin L (CTSL), cellular proteases involved in SARS-CoV-2 spike protein activation, are largely unexplored in most species. Here, we generated primary cell cultures from the respiratory tract of domestic and wildlife animals to assess their susceptibility to SARS-CoV-2 infection. Additionally, the presence of *ACE2*, *TMPRSS2* and *CTSL* within respiratory tract compartments was investigated in a range of animals, some with unknown susceptibility to SARS-CoV-2. Productive viral replication was observed in the nasal mucosa explants and precision-cut lung slices from dogs and hamsters, whereas culture models from ferrets and multiple ungulate species were non-permissive to infection. Overall, whereas *TMPRSS2* and *CTSL* were equally expressed in the respiratory tract, the expression levels of *ACE2* were more variable, suggesting that a restricted availability of ACE2 may contribute to reduced susceptibility. Summarized, the experimental infection of primary respiratory tract cell cultures, as well as an analysis of entry-factor distribution, enable screening for SARS-CoV-2 animal reservoirs.

## 1. Introduction

Beta-coronaviruses have been the origin of three major zoonotic respiratory disease outbreaks in the last two decades: Severe acute respiratory syndrome coronavirus 2 (SARS-CoV-2) as the causative agent of coronavirus disease 2019 (COVID-19) was discovered most recently [1], whereas Severe acute respiratory syndrome coronavirus (SARS-CoV) and Middle East respiratory syndrome coronavirus (MERS-CoV) emerged in 2002 and 2012, respectively [2,3,4]. Progenitors of SARS- and MERS-CoV have been detected in bats, providing strong evidence that both viruses emerged from chiropteran hosts [5,6,7]. Further, the zoonotic transmission involved palm civets for SARS-CoV and dromedary camels for MERS-CoV as intermediate hosts [6,8,9]. The identification of non-human sources of SARS-CoV-2 is an important task, but so far, limited progress has been made. Bats and pangolins harbor viruses closely related to SARS-CoV-2 and are therefore discussed as reservoirs for the progenitor virus of SARS-CoV-2 [1,10,11,12]. However, bat species or pangolins harboring the direct progenitor remain to be identified, and the search for potential intermediate hosts responsible for transmitting the virus into the human population is still ongoing [1,13,14]. Natural SARS-CoV-2 infection of numerous animal species, both domestic (e.g., cats, dogs, mustelids) [15] and wildlife, living in captivity (predatory cats, otters, gorillas) [16] as well as free-ranging (white-tailed deer) [17], have been reported. Cervidae are currently of particular interest, with recent studies reporting a high seroprevalence in wild white-tailed deer in the USA [17,18]. Based on phylogenetic analyses, it has been shown that several intraspecies transmission events took place among deer, resulting in viral mutations that are not frequently found in human SARS-CoV-2 isolates [17]. Such mutations arising in animal hosts may affect receptor usage—especially those mutations that occur within the receptor binding domain (RBD)—or modulate the efficiency of antibody-mediated neutralization [19,20,21]. For mink, another highly susceptible species for SARS-CoV-2 infection, it has been reported that zoonotic transmission of SARS-CoV-2 from infected mink to humans and vice versa occurred [22], raising awareness for the urgent need of identifying potential intermediate and reservoir hosts of SARS-CoV-2. Following experimental infection, non-human primates [23], ferrets [24], hamsters [25], fruit bats [26], raccoon dogs [27], bank voles [28], rabbits [29], tree shrews [30], deer mice, bushy-tailed woodrats and striped skunks [31] have been discovered to be susceptible to SARS-CoV-2, although not all of them exhibit clinical signs of infection, therefore limiting their value as animal models. However, there is still a substantial lack of knowledge regarding the susceptibility of numerous animal species, especially wildlife animals. For many of these species, experimental infection studies are difficult to conduct due to the limited availability of animals, some of which are listed as endangered species, difficulties in the handling of certain species under laboratory conditions and ethical considerations. The use of primary cells from freshly deceased animals enables an in-depth in vitro/ex vivo analysis of the virus–host interaction within the respiratory tract from various species and is in agreement with the 3R principles to replace, reduce and refine animal experimentation that are required by European legislation (Directive 2010/63/EU).

The entry process of SARS-CoV-2 is mediated by the viral spike protein (S), which consists of two subunits. The surface subunit S1 comprises the RBD, which facilitates viral attachment to the surface of target cells. Similar to SARS-CoV, it has been shown that SARS-CoV-2 S uses angiotensin-converting enzyme 2 (ACE2) as the receptor for cell entry [32]. The S2 subunit of SARS-CoV-2 S facilitates the fusion of the viral and cellular membrane to release the viral genome into the cytoplasm of target cells. This process requires priming of the S by cellular proteases at the S1/S2 and the S2′ site [33,34]. Whereas the S1/S2 boundary is cleaved by furin within the virus-producing cell, cleavage at the S2′ site occurs in the target cell and can either be facilitated by transmembrane protease serine subtype 2 (TMPRSS2) at the cell surface or by cathepsin L (CTSL) within the endosomal compartment [32,35,36,37]. The varying availability of ACE2 in the target organs of different species seems to be linked to differences in the susceptibility to and tissue tropism of SARS-CoV-2 [38,39,40,41]. While for some animal species, such as felids, pigs, cattle, sheep, hamsters, mink, mice and ferrets, the organ expression of ACE2 has been investigated [38,42,43,44], the expression pattern of other crucial host factors, especially in the different regions of the respiratory tract, is currently mostly unknown. 

In this study, we investigated the mRNA expression of *ACE2*, *TMPRSS2* and *CTSL* in the upper and lower respiratory tracts of various animal species in order to determine tissue- and species-specific differences in the expression levels of cellular factors required for SARS-CoV-2 entry. Furthermore, we analyzed the SARS-CoV-2 infection of nasal mucosa explants (NMEs), tracheal epithelial cells cultured at an air–liquid interface (ALI) and precision-cut lung slices (PCLSs) from the native tissues of domestic and wildlife animals, as well as species commonly used as animal models for SARS-CoV-2 infection. The infection of human samples was analyzed as a reference. Infected primary cell cultures were examined for viral replication, virus antigen expression and associated cytopathic effects.

## 2. Materials and Methods

### 2.1. Collection of Samples

The nasal mucosa, trachea and lungs used for the establishment of primary cell cultures derived from a total of 11 animal species, including dogs, ferrets, pigs, cattle and hamsters, and one mouflon, moose, nyala, giraffe, camel and alpaca, respectively, free of respiratory diseases. Tissue samples were collected during routine diagnostic necropsies in the Department of Pathology, University of Veterinary Medicine, Hanover (dogs, pigs, cattle, mouflon, moose, nyala, giraffe, camel, alpaca), from a local slaughterhouse (pigs) or from control animals used in experimental studies (ferrets, hamsters), which were performed in strict accordance with the guidelines of German animal protection law and were approved by the relevant German authority (the local authority approval number: COVID-19 pathogenesis 33.19-42502-04-20/3402, TiHo-T-2021-11 B_MvKB). Sampling was dependent on tissue availability and preservation; therefore, samples were not always taken from each location in each animal (Table 1). In addition, lung tissue from human patients who underwent lobe resection at the Hannover Medical School (MHH) was used to generate PCLSs. The experiments with human lung tissue were approved by the Ethics Committee of the Hannover Medical School (MHH, Hannover, Germany) and are in compliance with the Code of Ethics of the World Medical Association (number 2701-2015). All patients or their next of kin gave written informed consent for the use of their lung tissue for research. 

### 2.2. Generation of Primary Cell Cultures 

The generation of NMEs has been previously described [45]. Briefly, the mucosa was separated from the nasal septum, rinsed with a tissue-friendly disinfecting solution (Prontosan C; B. Braun, Melsungen, Germany) and phosphate-buffered saline (PBS, Sigma-Aldrich, Darmstadt, Germany), then divided into approximately 20 mm^2^ sized rectangles and, with the epithelium facing upwards, transferred onto fine-meshed membranes (pore size: 0.4 µm; VWR) supported by a transwell system (Sarstedt, Nümbrecht, Germany). The NMEs were cultured under ALI conditions with serum-free Dulbecco’s Modified Eagle’s Medium (DMEM; Gibco, Thermo Fisher Scientific, Waltham, USA) and added penicillin–streptomycin (10000 U/mL penicillin, 10 mg/mL streptomycin; Sigma-Aldrich, Darmstadt, Germany), enrofloxacin (50 mg/mL; Bayer, Leverkusen, Germany) and amphotericin B (250 µg/mL; Sigma-Aldrich, Darmstadt, Germany) at 37 °C with 5% CO_2_. 

The primary tracheal epithelial cells cultured at ALI conditions were generated as previously described [45]. Briefly, the dissected trachea was washed with PBS (Sigma-Aldrich, Darmstadt, Germany), followed by enzymatic digestion with 1 mg/mL protease (Sigma-Aldrich, Darmstadt, Germany) and 0.01 mg/mL desoxyribonuclease I (Sigma-Aldrich, Darmstadt, Germany) for 24 h at 4 °C. After separating the epithelial cells from the tissue, they were transferred onto type I collagen (Sigma-Aldrich, Darmstadt, Germany) coated flasks and cultured at 37 °C with 5% CO_2_ until reaching 70–80% confluence. The cells were harvested and seeded onto type IV collagen (Sigma-Aldrich, Darmstadt, Germany) coated, semipermeable transwell membranes (pore size: 0.4 µm; VWR, Darmstadt, Germany) at a density of 0.35 × 10^6^ cells per membrane, with 250 µL ALI medium consisting of 50% DMEM (Gibco, Thermo Fisher Scientific, Waltham, USA), 50% Bronchial Epithelial Cell Growth Basal Medium (Clonetics, Basel, Switzerland) and additives as previously described [42]. Another 500 µL of ALI medium was added to the basolateral compartment, and the cultures were maintained at 37 °C with 5% CO_2_. Regular measurements of transendothelial/epithelial resistance (TEER) using a voltmeter (Millicell ERS-2 system MERS 00002, Merck, Darmstadt, Germany) were performed to determine cellular coherence, and after one week in culture, ALI conditions were initiated. Cellular differentiation was determined to be complete after three weeks in ALI condition.

Animal- [45] and human-derived [46] PCLSs were generated as previously described. Briefly, the individual lung lobes were filled with Low Melting Agarose (Gerbu, Heidelberg, Germany) dissolved in RPMI medium (Thermo Fisher Scientific, Waltham, USA) at 37 °C. After cooling, they were cut into small, cylinder-shaped pieces with 8 mm or 3 mm in diameter, depending on the species, using a coring press (Alabama R&D, Munford, USA) and separated into equally thick (250 µm) slices by a Krumdieck tissue slicer (Alabama R&D, Munford, USA). After washing the PCLSs three times with DMEM/F12 (without phenol red, Gibco, Thermo Fisher Scientific, Waltham, USA) with 1% penicillin–streptomycin, 50 mg/mL enrofloxacin and 250 µg/mL amphotericin, they were transferred to 24-transwell cell culture plates (Nunc, Thermo Fisher Scientific, Waltham, USA) containing 1000 µL of the same medium and maintained at 37 °C with 5% CO_2_. By semi-quantitatively observing the movement of the ciliated cells of the bronchi and bronchioles at 200× magnification via light microscopy (Olympus IX-70, Olympus Optical Co. GmbH, Hamburg, Germany), the vitality of the cultured tissue was ensured. 

### 2.3. Viral Growth Kinetics of SARS-CoV-2 Infected Primary Respiratory Cell Cultures 

All infection experiments with SARS-CoV-2 were conducted under biosafety level 3 conditions at the Infection Biology Unit, German Primate Center, Leibniz Institute for Primate Research, Göttingen, Germany. The infection of primary cell cultures was performed as previously described [42]. Briefly, NMEs and ALI cultures were infected from the apical side with 1 × 10^4^ infectious particles of SARS-CoV-2 isolate NK, Pango lineage B.1.513 (provided by Stephan Ludwig, Institute of Virology, University of Münster), in an inoculation volume of 100 µL. After 1 h of incubation, the inoculum was removed and the cells were washed three times with PBS from both sides, before 500 µL of fresh medium was added to the basal side. Newly released viral particles were harvested from the apical side at 1 h post infection (p.i.) and on a daily basis by incubation with 100 µL cell culture medium at 37 °C for 10 min. The transwell membranes were rinsed with the medium three times and the virus-containing supernatants were collected and stored at −80 °C until further usage. The PCLSs were infected with 1 × 10^5^ infectious viral particles of SARS-CoV-2 isolate NK in an inoculation volume of 500 µL. The supernatants were collected at 1 h and 1–4 days p.i. by harvesting and subsequent replacement of 100 µL of culture medium. The ciliary activity of infected and uninfected PCLSs was determined prior to and post infection every 24 h by light microscopy using a Keyence BZ-X800 microscope (Keyence, Neu-Isenburg, Germany). Viral titers were determined by titration of 10-fold serial dilutions of SARS-CoV-2 containing supernatants on Vero E6 cells, followed by agarose overlay. The supernatants of uninfected cell cultures were harvested and titrated accordingly to exclude the occurrence of unspecific cytopathic effects. At 3 days p.i., virus-induced plaques were counted and multiplied with the reciprocal of the dilution factor, and the volume used for the infection of Vero E6 cells. Viral titers are given as plaque-forming units/mL.

### 2.4. Detection of ACE2, TMPRSS2 and CTSL mRNA by qPCR 

With masses of 50–100 mg, organ samples derived from the nasal mucosa, trachea, lung and kidney of various mammalian animal species, including felids (cat, lion, cheetah, lynx), carnivores (dog, golden jackal, ferret, raccoon, red panda), lagomorphs (rabbit), primates (orangutan), odd-toed ungulates (horse) and even-toed ungulates (pig, wild boar, cattle, sheep, goat, nyala, moose, giraffe, camel, alpaca) (Supplementary Materials Tables S1–S3), were mechanically disrupted by cutting, followed by incubation with 1 mL TRIzol reagent (Invitrogen, Thermo Fisher Scientific, Waltham, USA) for 5 min at room temperature. Next, the samples were homogenized using a bead-beating tissue homogenizer, and RNA was extracted using TRIzol reagent. cDNA was generated from the RNA samples using the SuperScript III First-Strand Synthesis System and random hexamers (Thermo Fisher Scientific, Waltham, USA). Next, the cDNA was subjected to SYBR Green qPCR (Thermo Fisher Scientific, Waltham, USA), targeting *ACE2*, *TMPRSS2* or *CTSL*. The primers used for qPCR and the species-specific sequences of the targets are given in Supplementary Materials Table S4. Cycle threshold (ct) values were normalized to total RNA. Dilution series of expression plasmids containing cat ACE2 [42], pig ACE2, human TMPRSS2 [47] and human CTSL [48] were used as standards to calculate the amount of genomic equivalence (GE) based on the ct values.

### 2.5. Light Microscopic Evaluation of Primary Cell Cultures

The SARS-CoV-2 infected NMEs, ALI cultures and PCLSs, as well as the uninfected controls, were washed three times at 1–5 days p.i. with PBS and fixed in 10% formalin for 24 h. The formalin-fixed samples were embedded in paraffin wax, and 2 μm thick serial sections were generated and stained with hematoxylin and eosin (H&E) or used for immunohistochemical and immunofluorescence staining. 

The H&E-stained sections, derived from cell cultures showing detectable replication of SARS-CoV-2, were evaluated for virus-induced cytopathic effects with the criteria listed in Supplementary Materials Table S5, with the exception of hamster NMEs, which could not be further analyzed because of the limited number of samples available.

### 2.6. Immunohistochemical and Immunofluorescence Analysis 

An immunohistochemical analysis was conducted on formalin-fixed, paraffin-embedded (FFPE) samples, using only sections derived from the samples with confirmed SARS-CoV-2 replication, by applying the avidin–biotin–peroxidase complex (ABC; Vector Laboratories, Burlingame, USA) method as previously described [49,50]. The investigated antibodies were caspase-3, CD204 (not performed in ALI cultures), α-tubulin and Ki67. The reaction was carried out overnight at 4 °C. All details regarding the aforementioned antibodies are listed in Supplementary Materials Table S6. For negative controls, the specific primary antibodies were replaced by ascitic fluid from non-immunized BALB/cJ mice (for CD204, α-tubulin, Ki67) and serum from non-immunized rabbits (caspase-3). The dilution of the negative controls was chosen according to the protein concentration of the replaced primary antibodies. For the evaluation of ALI cultures, the number of positively stained cells for each high-power field (HPF, 400× magnification) was counted and compared with the total number of cells, which was counted for each slide in the H&E staining. For the NMEs and PCLSs, all cells with positive immunoreactivity for caspase-3, CD204 and Ki67 were counted in five random HPFs (400× magnification), including parenchyma and stroma. The number of ciliated epithelial cells labeled by α-tubulin in NMEs and PCLSs was divided by the length of the bronchial or nasal epithelium in HPFs (400× magnification), respectively.

Immunofluorescence labeling for the SARS-CoV-2 nucleoprotein (NP) was also carried out on SARS-CoV-2 positive FFPE samples as previously described [51], with minor variations. Briefly, the FFPE tissue sections were deparaffinized, rehydrated and pre-treated for antigen retrieval. Following blocking of nonspecific bindings with goat serum, the sections were incubated with the primary and secondary antibody for 90 and 60 min at room temperature, respectively. All details regarding the aforementioned antibody are listed in Supplementary Materials Table S6. For negative controls, the specific primary antibodies were replaced by ascitic fluid from non-immunized BALB/cJ mice. The dilution of the negative control was chosen according to the protein concentration of the replaced primary antibody. Fluoroshield^TM^ mounting medium (Sigma-Aldrich, Darmstadt, Germany) was used on slides for the staining of cell nuclei and the mounting of coverslips. The sections were screened for the expression of the SARS-CoV-2 NP with a fluorescence microscope (BZ-9000E microscope, Keyence, Neu-Isenburg, Germany). All samples were evaluated by using 40×, 100× and 400× magnifications.

### 2.7. Statistical Evaluation 

A statistical analysis was conducted using SPSS software (IBM SPSS Statistics 26; IBM, Armonk, USA). Shapiro–Wilk normality tests, followed by Mann–Whitney U tests, were performed to analyze the results from the light microscopic (Supplementary Materials Table S7) and immunohistochemical (Supplementary Materials Table S8) evaluations. As detected by Mann–Whitney U tests, differences between groups were considered significant at a *p*-value of less than 0.05. Graphics from the obtained statistical data were created using GraphPad Software (GraphPad Prism, version 9.1.0, La Jolla, CA, USA). 

## 3. Results

### 3.1. mRNA Expression of ACE2, TMPRSS2 and CTSL in the Upper and Lower Respiratory Tract of Animal Species

**Figure 1:**
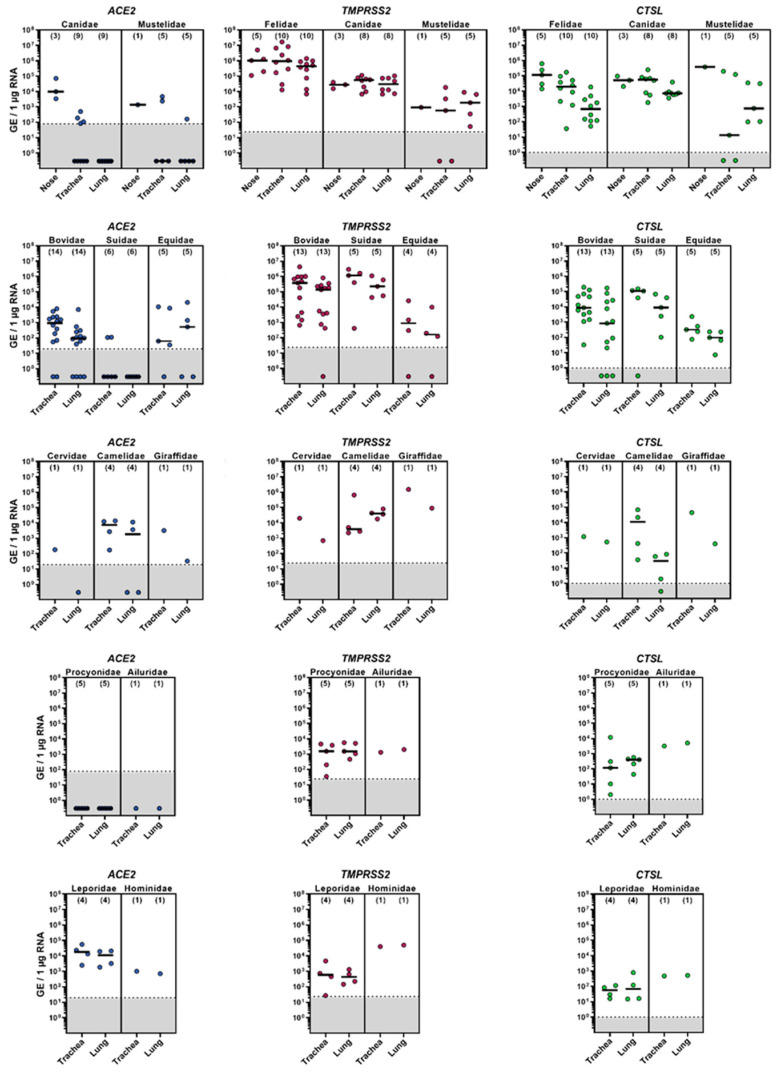
mRNA expression of *ACE2*, *TMPRSS2* and *CTSL* in the respiratory tract of domestic and wild animals. RNA was isolated from the nasal mucosa, trachea and lungs and subjected to RT-PCR, followed by quantitative PCR, targeting *ACE2*, *TMPRSS2* or *CTSL*. Dilution series of expression plasmids containing the respective targets were used to generate standard curves to calculate the amounts of genomic equivalence (GE) based on the ct values. The graphs show average (median, indicated by black lines) data from individual animals (n is indicated by numbers in brackets). For each individual sample (circle) five technical replicates were measured, and the average (mean) is shown. Samples within the gray shaded area were below the threshold (determined separately for each primer pair).

The mRNA levels of cellular factors crucial for SARS-CoV-2 entry—including ACE2, TMPRSS2 and CTSL—were determined for the nasal mucosa, trachea and lungs of a variety of mammalian species belonging to the families Canidae, Mustelidae, Felidae, Bovidae, Suidae, Equidae, Cervidae, Camelidae, Giraffidae, Procyonidae, Ailuridae, Leporidae and Hominidae (Figure 1). *ACE2* was frequently detected in the nasal mucosa of carnivores, as well as in the trachea and lungs of some ungulates, especially bovines, and rabbits (Supplementary Materials Table S1). In contrast, no or only limited *ACE2* expression was observed in the lung tissue derived from carnivores and pigs. Generally, *TMPRSS2* and *CTSL* were detected at high copy numbers of up to 10^7^ genomic equivalents (GE)/µg RNA and exceeded those of *ACE2* for all species except those of the family Leporidae (Supplementary Materials Tables S2 and S3). The expression of *TMPRSS2* within the different regions of the respiratory tract was equally distributed for all species, whereas for *CTSL*, the expression levels differed among species, displaying higher amounts of *CTSL* mRNA in the nasal mucosa and trachea compared to lung tissue. 

### 3.2. SARS-CoV-2 Susceptibility of Various Animal Species Determined by Three Primary Cell Culture Models

**Figure 2:**
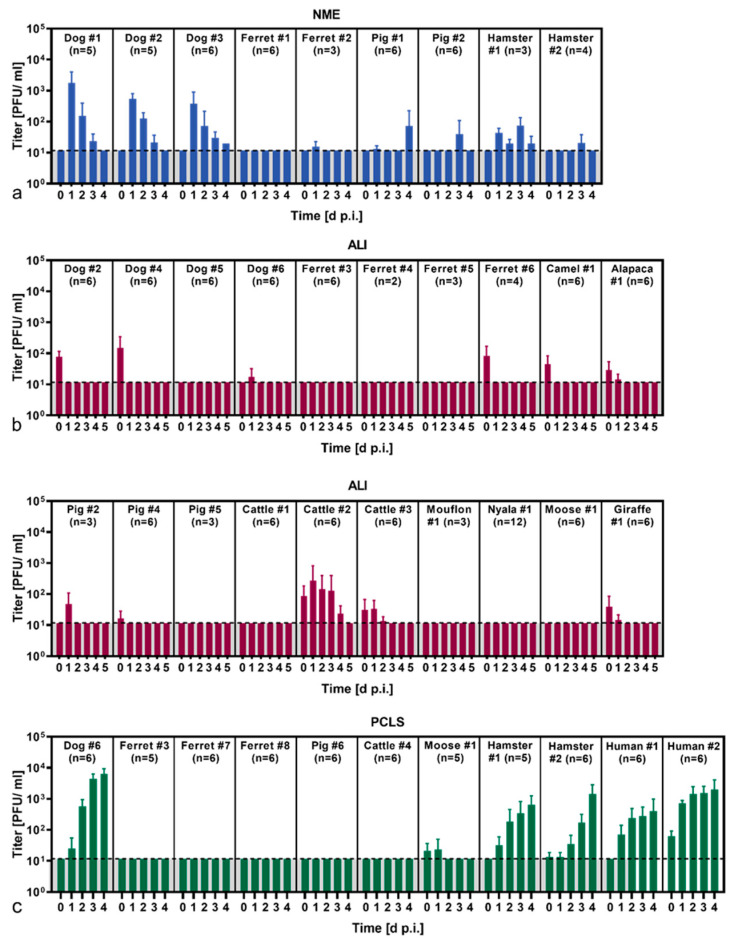
Viral replication of SARS-CoV-2 in primary respiratory cell cultures: (**a**) Nasal mucosa explants (NME), (**b**) air–liquid interface (ALI) cultures generated from tracheal epithelial cells and (**c**) precision-cut lung slices (PCLS) were infected with SARS-CoV-2. The supernatants were collected at the indicated time points (days post infection, d p.i.), and viral titers were determined by titration on Vero E6 cells. Viral titers are given as plaque-forming units (PFU)/mL. The graphs show means and standard deviation of n replicates. The dashed lines indicate the limit of detection (=11.76 PFU/mL).

In vitro and ex vivo primary cell culture models generated from the upper and/or lower respiratory tract were applied to investigate the susceptibility of several animal species to SARS-CoV-2 infection (Table 1). Productive viral replication of SARS-CoV-2, Pango lineage B.1.513, was observed in dog NMEs characterized by a rapid increase in viral titers within 24 h p.i. (Figure 2a). A slight increase in viral titers was obtained in the NMEs from hamsters, whereas no efficient replication was detected in the NMEs derived from ferrets and pigs (Figure 2a). Tracheal ALI cultures derived from dogs, ferrets, pigs and various ungulate species did not support SARS-CoV-2 replication (Figure 2b). Notably, efficient viral replication was detected in one bovine tracheal ALI culture at 24 h p.i. (Figure 2b). Regarding PCLSs, similar viral growth kinetics were observed for dog, hamster and human primary lung cell cultures, reaching peak titers at day 4 p.i. (Figure 2c). The PCLSs derived from ferrets, a pig, a cow and a moose did not support viral replication. The ciliary activity of the PCLSs was not affected by SARS-CoV-2 infection, as no differences between the infected and uninfected cultures were observed (Supplementary Materials Figure S1).

### 3.3. Cytopathic Features of Primary Cell Cultures Exposed to SARS-CoV-2 Infection

The light microscopic evaluation of H&E-stained NMEs, ALI cultures and PCLSs of the animal species that supported SARS-CoV-2 replication revealed organotypic structures of all applied culture systems. There were mild variations regarding the severity of intracytoplasmic vacuolization, nuclear degeneration and loss of cilia between individual uninfected and infected samples of all three culture systems. A multifocal loss of cilia, perivascular edema, as well as small numbers of vacuoles in interstitial cells were observed in the infected and uninfected NMEs and PCLSs from hamsters, humans and dogs. Bovine-derived ALI cultures showed a formation of large vacuoles with clusters of apoptotic cells in both the infected and uninfected cultures. Overall, the light microscopic evaluation of SARS-CoV-2 infected primary cell cultures showed few statistically significant differences compared to the respective uninfected control samples (Supplementary Materials Table S7). Notably, SARS-CoV-2 infected human PCLSs displayed foci of cells, presumably type II pneumocytes, with plumped cytoplasm, interpreted as type II pneumocyte hypertrophy.

### 3.4. Detection of SARS-CoV-2 Antigen, Cellular Tropism and Immunolabeling of Relevant Cellular Markers

**Figure 3:**
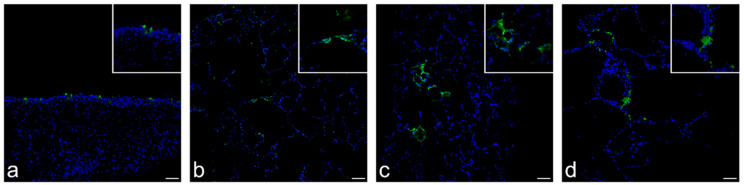
Detection of the SARS-CoV-2 nucleoprotein (NP) in primary respiratory cell cultures derived from a dog, hamster and human by immunofluorescence. In paraffin-fixed tissue sections of SARS-CoV-2 infected primary respiratory tract cell cultures, immunofluorescence staining of SARS-CoV-2 NP (green) and nuclei (blue) was performed. (**a**) Respiratory epithelium and submucosal glands of canine nasal mucosa explants. (**b**) Canine, (**c**) hamster and (**d**) human precision-cut lung slices with alveolar and bronchial epithelium as well as connective tissue (100× magnification). Inset shows positive signal at higher magnification (400× magnification). Scale bar represents 50 µM.

NMEs (dog), ALI cultures (cow) and PCLSs (dog, hamster and human) were investigated by immunofluorescence for the presence of the SARS-CoV-2 NP. In the canine NMEs, the viral antigen was detected at 1 and 2 days p.i., with positive cells multifocally localized in the respiratory epithelium, as well as in submucosal glands (Figure 3a). No viral NP was detected in bovine ALI cultures, although viral replication was measured. Immunolabeling of canine, hamster and human PCLSs revealed SARS-CoV-2 NP positive cells at 4 days p.i., 3 and 4 days p.i., and 2, 3, and 4 days p.i., respectively. SARS-CoV-2 infected PCLSs showed focal areas with SARS-CoV-2 NP immunolabeled cells, with the primary infected cell type presumably being type I and occasionally type II pneumocytes (Figure 3b–d).

Immunohistochemistry using antibodies for the detection of cilia (α-tubulin), cell death (caspase-3), macrophages (CD204) and proliferation of cells (Ki67) was performed to further characterize cell cultures, which allowed for viral replication. All evaluated samples showed positive immunolabeled cells for the aforementioned markers in both the infected and uninfected samples, showing very few significant differences (Supplementary Materials Table S8), likely not due to viral infection but to individual variations of the investigated cultures.

## 4. Discussion

Here, we report the distribution of *ACE2*, *TMPRSS2* and *CTSL*, crucial factors for SARS-CoV-2 entry, in the respiratory tract of numerous animal species. Additionally, we generated and analyzed in vitro and ex vivo primary cell culture models as screening tools for species susceptibility to SARS-CoV-2 infection. The NMEs and PCLSs from dogs, hamsters and humans supported replication of SARS-CoV-2, whereas the ALI cultures from various ungulates, including pigs, mouflon, nyala, camel, alpaca, moose and giraffe did not support viral replication, with the exception of the tracheal epithelial cells derived from a cow, providing further insight into the natural host spectrum of this highly relevant virus. Further, our results emphasize the usefulness of primary cell culture models for SARS-CoV-2 research.

The process of viral entry into host cells is the first step of the coronavirus replication cycle, representing a barrier that the virus needs to overcome when infecting new host species. For SARS-CoV-2, ACE2 has been shown to serve as the main cellular receptor [1,32]. In order to predict the susceptibility of domestic and wildlife animals to SARS-CoV-2 based on species-specific ACE2 protein sequences, large-scale in silico sequence analyses have been performed [52,53,54,55]. Such analyses were completed by functional studies showing that a broad range of animal ACE2 orthologues serve as receptors for SARS-CoV-2 [56,57]. Based on these data, it may be assumed that closely related species share highly conserved ACE2 orthologues that exhibit a similar binding efficiency.

Furthermore, besides compatibility, the availability of ACE2 plays an important role in determining the cell and host tropism of SARS-CoV-2. In addition to ACE2, the cellular proteases TMPRSS2 and CTSL are also important for SARS-CoV-2 entry, as they are responsible for the proteolytic activation of SARS-CoV-2 S [32]. Here, we investigated the mRNA expression levels of *ACE2*, as well as *TMPRSS2* and *CTSL*, in different regions of the respiratory tracts of various mammalian species. In general, the expression levels of *ACE2* were more variable compared to those of *TMPRSS2* and *CTSL* with regard to species- and tissue-specific differences, with the lowest amounts of *ACE2* detected in lung tissue. Similar observations have also been reported for humans [58], suggesting that the availability of the receptor ACE2, rather than TMPRSS2 or CTSL, may contribute to SARS-CoV-2 susceptibility of potential target cells.

The infection of primary cell cultures, overall, revealed that NMEs and PCLSs derived from dogs support efficient SARS-CoV-2 replication, indicating that canines are permissive to viral infection. This is consistent with previous studies confirming the susceptibility of canines to SARS-CoV-2 infections under experimental and natural conditions [59,60,61]. Based on in silico ACE2 sequence analyses [55] and functional studies showing that SARS-CoV-2 S exhibits a lower affinity to dog as compared to human ACE2 [62], it has been suggested that canines have a lower susceptibility to SARS-CoV-2 infections compared to other mammalian species. However, several seroepidemiological studies focusing on dogs living in COVID-19 positive households indicate that the transmission rate from infected owners to their canine pets may be higher than expected [63,64]. 

In the present study, high mRNA expression levels of *ACE2* were found in the nasal mucosa samples of dogs, consistent with another study, where substantial amounts of ACE2 were detected in canine nasal cavity explants [65]. This finding, in combination with the obtained rapid and highly efficient replication in NMEs, implies that the nose may constitute the primary site of viral entry and replication of SARS-CoV-2 in canines. Previous in vivo studies report that viral RNA was frequently found in canine nasal swabs, thereby supporting our hypothesis [66]. The fact that PCLSs derived from dogs in the present study were susceptible to infection, even though *ACE2* expression levels were low in lung tissue samples, raises the question whether ACE2-independent entry occurred. It has recently been shown that additional cellular receptors, including neuropilin, ASGR1 and KREMEN1, may support SARS-CoV-2 attachment and entry [67,68]. So far, there is no information about the role of these alternative receptors for SARS-CoV-2 in mammalian species other than humans.

Hamsters and ferrets are some of the most frequently used animal species for SARS-CoV-2 experimental studies [24,25,69,70,71]. Hamster-derived PCLSs generated for this study supported viral entry and replication, similar to previous reports [72]. Surprisingly, only the nasal mucosa samples from one out of two hamsters were permissive to SARS-CoV-2 infection, whereas in in vivo studies with hamsters, the nasal mucosa was highly susceptible [25,73,74]. This could be due to suboptimal culture conditions or insufficient numbers of target cells due to the small size of the cultures. In contrast, none of the primary cell culture models derived from ferrets permitted efficient SARS-CoV-2 replication in this study. As a possible explanation, the viral loads in experimentally infected ferrets have been shown to be the highest in the upper respiratory tract, including the nose and throat [26,61,75,76]. In some experiments, viral RNA was mostly or only detected in the nasal cavity, with little or no detection of the virus in the lower respiratory tract [26,61,77]. More precisely, in one study using a light sheet microscopy-assisted 3D analysis method to study in detail the viral antigen distribution in the upper and lower respiratory tract of ferrets, infection foci of SARS-CoV-2 were distributed in an oligofocal pattern, affecting mainly the dorsal nasal conchae [78]. Similar results were demonstrated in another experiment, where the SARS-CoV-2 NP was only detected in the respiratory epithelium of the rostral nasal conchae, with an absence of the viral antigen in all other examined organs [71]. The NMEs used in this study were generated from the middle and caudal region of the nasal mucosa associated with the nasal septum, since the achievable number of generated samples was deemed higher in these regions. Therefore, it seems likely that the lack of SARS-CoV-2 replication in NMEs results from inappropriate sampling regions of the nasal mucosa. 

Out of all primary cell cultures derived from ungulates, one tracheal epithelial ALI culture of a cow supported the efficient replication of SARS-CoV-2. Similarly, SARS-CoV-2 replication was seen in bovine and ovine tracheal and lung tissue explants in a previous study [43]. However, in the present study, the viral antigen was not detected by immunolabeling. The lack of antigen detection despite the observed replication could be due to overall low viral titers. This is consistent with a previous in vivo study, which revealed the low susceptibility of cattle to SARS-CoV-2 following experimental infection [79]. It is of note that a recent study reported SARS-CoV-2 seropositivity in cattle from different farms in Germany, suggesting that, although the overall susceptibility of cattle seems to be low, spillover events are still possible in this species [80]. 

As a limitation, in the present study, the light microscopic evaluation of all applied cell culture models revealed no virus-related cytopathic effects, including the loss of cilia. Some statistically significant differences between the infected and uninfected samples were obtained as a result of the evaluation of H&E and immunohistochemical staining. However, these differences were interpreted as artifacts due to culture conditions, affecting both infected and uninfected cultures, and/or individual variations and their biological significance is questionable. This indicates that even though ALI cultures, NMEs and PCLSs have been established as primary cell culture models for infection studies and can support SARS-CoV-2 replication, limiting factors, such as a disadvantageous environment, missing cell–cell interactions or the lack of a complete immune response, may restrict the ability to draw conclusions regarding cytopathogenic effects caused by viral infection when examining these cultures.

Overall, regarding animals and SARS-CoV-2, there are still many unknown aspects, such as the identity of the intermediate host responsible for introducing the virus into the human population or the quantity of undiscovered animal reservoirs that may uphold recirculation of new virus variants. Already, large-scale in silico phylogenetic analyses have been employed to determine the susceptibility of related animal species based on the comparison of sequence identity and expression patterns of crucial entry receptors [55], and numerous animal species have been experimentally infected with SARS-CoV-2 [81]. However, efforts to develop new techniques for the identification of SARS-CoV-2 host species should still be intensified, whilst taking into consideration the aim to circumvent in vivo animal experiments whenever possible, to gain further insight into the transmission dynamics and the reverse zoonotic potential of the virus.

## Figures and Tables

**Table 1:** Overview of primary cell cultures of the respiratory tract inoculated with SARS-CoV-2.

Family	Species	ID/(Internal Identification Number)	Culture System	Number of Infected Cultures	Number of Uninfected Controls
Canidae	Dog (*Canis lupus familiaris*)	Dog #1 (S656/21)	NME	5	2
		Dog #2 (S773/21)	NME	5	2
		Dog #3 (S947/21)	NME	6	2
		Dog #2 (S773/21)	ALI	6	2
		Dog #4 (S433/21)	ALI	6	3
		Dog #5 (S546/21)	ALI	6	3
		Dog #6 (S582/21)	ALI	6	3
		Dog #6 (S582/21)	PCLS	6	3
Mustelidae	Ferret (*Mustela putorius furo*)	Ferret #1 (V385/20)	NME	6	3
		Ferret #2 (S944/21)	NME	3	1
		Ferret #3 (V713/21)	ALI	6	2
		Ferret #4 (V749/21)	ALI	2	1
		Ferret #5 (V385/21)	ALI	4	2
		Ferret #6 (V919/21)	ALI	4	1
		Ferret #3 (V713/21)	PCLS	5	4
		Ferret #6 (V919/21)	PCLS	6	5
		Ferret #7 (V728/20)	PCLS	6	4
Suidae	Pig (*Sus scrofa domesticus*)	Pig #1 (S948/21)	NME	6	2
		Pig #2 (S1002/21)	NME	6	2
		Pig #3 (S441/20)	ALI	3	0
		Pig #4 (S442/20)	ALI	6	3
		Pig #5 (S444/20)	ALI	3	3
		Pig #6 (S711/20)	PCLS	6	2
Bovidae	Cattle (*Bos taurus*)	Cattle #1 (S516/20)	ALI	6	3
		Cattle #2 (S657/20)	ALI	6	2
		Cattle #3 (S680/20)	ALI	6	2
		Cattle #4 (S765/20)	PCLS	6	3
	Mouflon (*Ovis aries musimon*)	Mouflon #1 (S407/20)	ALI	6	2
	Nyala (*Tragelaphus angasii*)	Nyala #1 (S601/20)	ALI	12	4
Camelidae	Camel (*Camelus bactrianus*)	Camel #1 (S747/20)	ALI	6	2
	Alpaca (*Vicugna pacos*)	Alpaca #1 (S758/20)	ALI	6	2
Cervidae	Moose (*Alces alces*)	Moose #1 (S612/20)	ALI	6	3
		Moose #1 (S612/20)	PCLS	5	2
Giraffidae	Giraffe (*Giraffa* sp.)	Giraffe #1 (S755/20)	ALI	6	2
Cricetidae	Hamster (*Mesocricetus auratus*)	Hamster #1 (S1038/20)	NME	3	1
		Hamster #2 (V84/21)	NME	4	2
		Hamster #1 (S1038/20)	PCLS	5	1
		Hamster #2 (V84/21)	PCLS	6	2
Hominidae	Human (*Homo sapiens*)	Human #1 (V386/20)	PCLS	6	3
		Human #2 (V387/20)	PCLS	6	3

Abbreviations: ALI, air-liquid interface cultures; NME, nasal mucosa explants; PCLS: precision-cut lung slices; SARS-CoV-2, Severe acute respiratory coronavirus 2.

## Data Availability

The data presented in this study are available on request from the corresponding author.

## Supplementary Materials

The following supporting information can be downloaded at: https://www.mdpi.com/article/10.3390/v14040828/s1. Figure S1: Ciliary activity of SARS-CoV-2 infected PCLS; Table S1: *ACE2* mRNA expression within the upper and lower respiratory tract of mammalian species; Table S2: *TMPRSS2* mRNA expression within the upper and lower respiratory tract of mammalian species; Table S3: *CTSL* mRNA expression within the upper and lower respiratory tract of mammalian species; Table S4: Sequences of primers used for qPCR and species-specific targets *ACE2*, *TMPRSS2* and *CTSL*; Table S5: Description of evaluation criteria of cytopathic features in culture systems used in the present study; Table S6: Primary antibodies used for immunofluorescence (IF) and immunohistochemistry (IHC); Table S7: Light microscopic evaluation of hematoxylin and eosin-stained culture systems; Table S8: Light microscopic evaluation of immunohistochemically stained culture systems.
