# Supplementary material for: Investigations on SARS-CoV-2 Susceptibility of Domestic and Wild Animals Using Primary Cell Culture Models Derived from the Upper and Lower Respiratory Tract
Source: Viruses. 2022 Apr 16;14(4):828. doi: 10.3390/v14040828 (PMC9032458; doi:10.3390/v14040828)
Supplement: Supplementary file 1 [file viruses-14-00828-s001.zip › viruses-1648544-supplementary.pdf]

Supplementary Material Figure S1. Ciliary activity of SARS-CoV-2 infected PCLS. Ciliary activity of infected (SARS-CoV-2, purple) and uninfected (mock, blue) PCLS was semi-quantitatively determined by light microscopy. The graphs show the means and SD of n replicates as indicated in each graph.

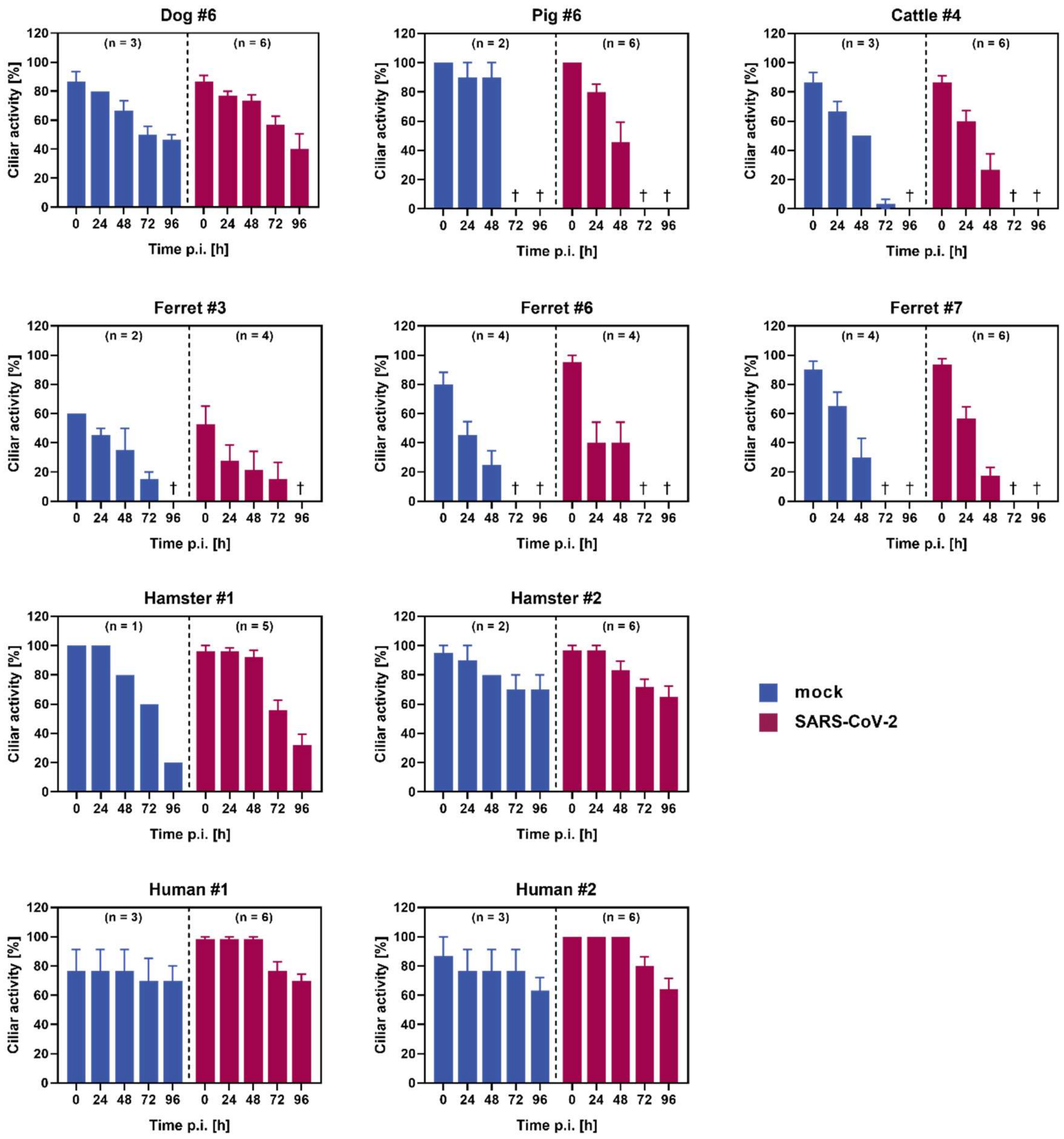

[1]Abbreviations: PCLS, precision-cut lung slices; p.i., post infection; SARS-CoV-2, severe acute respiratory syndrome coronavirus 2; SD, standard deviation;

Supplementary Material Table S1. *ACE2* mRNA expression within the upper and lower respiratory tract of mammalian species.

Family	Species	ID (Internal Identification Number)	Nasal mucosa	Trachea	Lung	Kidney
Canidae[1]	Dog (*Canis lupus familiaris*)	Dog #1 (S656/21)	71859	105	77	nd
		Dog #2 (S745/21)	3391	29	18	nd
		Dog #7 (S773/21)	9858	186	0	nd
		Dog #8 (S546/21)	n/a	493	1	nd
		Dog #9 (S585/20)	n/a	4	1	839
		Dog #10 (S624/20)	n/a	83	23	nd
		Dog #11 (S645/20)	n/a	60	8	nd
		Dog #12 (S690/20)	n/a	7	1	nd
	Golden Jackal (*Canis aureus*)	Jackal #1 (S542/20)	n/a	19	15	147
Mustelidae[1]	Ferret (*Mustela putorius furo*)	Ferret #1 (V385/20)	1369	2393	160	nd
		Ferret #3 (V713/20)	n/a	0	13	nd
		Ferret #4 (V749/20)	n/a	4652	22	nd
		Ferret #7 (V919/20)	n/a	6	22	nd
		Ferret #9 (V920/20)	n/a	23	8	nd
Procyonidae[1]	Raccoon (*Procyon lotor*)	Raccoon #1 (S651/20)	n/a	18	7	11369
		Raccoon #2 (S652/20)	n/a	26	2	nd
		Raccoon #3 (S656/20)	n/a	6	6	2183
		Raccoon #4 (S697/20)	n/a	28	2	nd
		Raccoon #5 (S775/20)	n/a	0	11	nd
Ailuridae[1]	Red Panda (*Ailurus fulgens*)	Red Panda #1 (S525/20)	n/a	21	25	38299
Leporidae[2]	Rabbit (*Oryctolagus cuniculus*)	Rabbit #1 (S715/20)	n/a	13361	19065	nd
		Rabbit #2 (S946/20)	n/a	2453	3163	nd

		Rabbit #3 (S1002/20)	n/a	22693	1849	nd
		Rabbit #4 (S1063/20)	n/a	54833	20601	nd
Suidae[2]	Pig (*Sus scrofa domesticus*)	Pig #7 (S620/20)	n/a	112	5	181
		Pig #8 (S674/20)	n/a	3	18	nd
		Pig #9 (S731/20)	n/a	8	5	234
		Pig #10 (S709/20)	n/a	14	6	nd
		Pig #11 (S856/20)	n/a	19	11	215
	Wild boar (*Sus scrofa*)	Wild boar #1 (S565/20)	n/a	109	19	568
Bovidae[2]	Cattle (*Bos taurus*)	Cattle #2 (S657/20)	n/a	19	131	nd
		Cattle #3 (S680/20)	n/a	6	0	nd
		Cattle #5 (S704/20)	n/a	671	108	nd
		Cattle #6 (S765/20)	n/a	71	86	nd
		Cattle #7 (S819/20)	n/a	191	36	nd
	Sheep (*Ovis aries*)	Sheep #1 (S687/20)	n/a	55	0	nd
		Sheep #2 (S720/20)	n/a	1557	9	nd
		Sheep #3 (S787/20)	n/a	1697	535	nd
		Sheep #4 (S809/20)	n/a	2252	61	nd
	Goat (*Capra hircus*)	Goat #1 (S602/20)	n/a	1215	3	nd
		Goat #2 (S644/20)	n/a	8001	248	nd
		Goat #3 (S722/20)	n/a	5360	0	nd
		Goat #4 (S788/20)	n/a	2143	7075	nd
	Nyala (*Tragelaphus angasii*)	Nyala #1 (S601/20)	n/a	386	305	256
Cervidae[2]	Moose (*Alces alces*)	Moose #1 (S612/20)	n/a	182	3	60
Giraffidae[2]	Giraffe (*Giraffa sp.*)	Giraffe #1 (S755/20)	n/a	3216	33	nd
Camelidae[2]	Camel (*Camelus bactrianus*)	Camel #1 (S747/20)	n/a	2719	1	61

	Alpaca (*Vicugna pacos*)	Alpaca #1 (S614/20)	n/a	173	4	nd
		Alpaca #2 (S615/20)	n/a	13644	11521	nd
		Alpaca #3 (S758/20)	n/a	12219	3667	nd
Equidae[2]	Horse (*Equus caballus*)	Horse #1 (S583/20)	n/a	8655	1387	nd
		Horse #2 (S617/20)	n/a	10615	20454	nd
		Horse #3 (S633/20)	n/a	7	13	nd
		Horse #4 (S649/20)	n/a	62	10	nd
		Horse #5 (S721/20)	n/a	35	522	nd
Homonidae[2]	Orangutan (*Pongo sp.*)	Orangutan #1 (S794/20)	n/a	999	693	nd

[1]*ACE2* mRNA expression is given as genomic equivalents (GE)/ 1 µg RNA. The table shows mean values for each animal and organ measured as quintuplicates. Threshold: primer pair 1: 78.69, primer pair 2: 19.6. Superscripted numbers indicate the primer pair used for qPCR.
[2]Abbreviations: ACE2, angiotensin-converting enzyme 2; n/a, no organ sample available; nd, not determined;

Supplementary Material Table S2. *TMPRSS2* mRNA expression within the upper and lower respiratory tract of mammalian species.

Family	Species	Sample ID	Nasal mucosa	Trachea	Lung
Felidae	Cat (*Felis catus*)	Cat #1 (S528/20)	n/a	167296	994160
		Cat #2 (S793/20)	n/a	1065216	873142
		Cat #3 (S933/20)	197321	28284	591099
		Cat #4 (S984/20)	1025943	290096	6970
		Cat #5 (S148/21)	110018	808028	265645
		Cat #6 (S169/21)	1279909	2318157	74213
	Lion (*Panthera leo*)	Lion #1 (S342/21)	5015021	6581201	377663
	Cheetah (*Acinonyx jubatus*)	Cheetah #1 (S640/20)	n/a	12708	13588
	Lynx (*Lynx sp.*)	Lynx #1 (S641/20)	n/a	16599720	1402511
		Lynx #2 (S817/20)	n/a	8208498	498033
Canidae	Dog (*Canis lupus familiaris*)	Dog #1 (S656/21)	39523	112688	7150
		Dog #2 (S745/21)	15245	63429	104924
		Dog #7 (S773/21)	27649	51456	6690
		Dog #8 (S546/20)	n/a	77364	47123
		Dog #10 (S624/20)	n/a	58445	12715
		Dog #11 (S645/20)	n/a	9592	13151
		Dog #12 (S690/20)	n/a	6732	73175
	Golden Jackal (*Canis aureus*)	Jackal #1 (S542/20)	n/a	19852	73445
Mustelidae	Ferret (*Mustela putorius furo*)	Ferret #1 (V385/20)	913	3314	6486
		Ferret #3 (V713/20)	n/a	4	1854
		Ferret #4 (V749/20)	n/a	18148	8761
		Ferret #7 (V919/20)	n/a	16	52
		Ferret #9 (V920/20)	n/a	579	346

Procyonidae	Raccoon (*Procyon lotor*)	Raccoon #1 (S651/20)	n/a	1532	1508
		Raccoon #2 (S652/20)	n/a	203	5064
		Raccoon #3 (S656/20)	n/a	34	460
		Raccoon #4 (S697/20)	n/a	4592	5630
		Raccoon #5 (S775/20)	n/a	3820	1080
Ailuridae	Red Panda (*Ailurus fulgens*)	Red Panda #1 (S525/20)	n/a	1312	2017
Leporidae	Rabbit (*Oryctolagus cuniculus*)	Rabbit #1 (S715/20)	n/a	4685	639
		Rabbit #2 (S946/20)	n/a	27	144
		Rabbit #3 (S1002/20)	n/a	741	222
		Rabbit #4 (S1063/20)	n/a	452	1301
Suidae	Pig (*Sus scrofa domesticus*)	Pig #8 (S674/20)	n/a	399757	42827
		Pig #9 (S731/20)	n/a	1174548	1096693
		Pig #10 (S709/20)	n/a	2939003	55449
		Pig #11 (S856/20)	n/a	414	227423
	Wild boar (*Sus scrofa*)	Wild boar #1 (S565/20)	n/a	1643524	600377
Bovidae	Cattle (*Bos taurus*)	Cattle #2 (S657/20)	n/a	668	4080
		Cattle #3 (S680/20)	n/a	2466	423
		Cattle #6 (S765/20)	n/a	40776	5564
		Cattle #7 (S819/20)	n/a	1446	3521
	Sheep (*Ovis aries*)	Sheep #1 (S687/20)	n/a	953944	137656
		Sheep #2 (S720/20)	n/a	686270	259142
		Sheep #3 (S787/20)	n/a	4280734	166232
		Sheep #4 (S809/20)	n/a	970327	224737
	Goat (*Capra hircus*)	Goat #1 (S602/20)	n/a	180736	181794
		Goat #2 (S644/20)	n/a	995039	356306

		Goat #3 (S722/20)	n/a	380286	24
		Goat #4 (S788/20)	n/a	480001	812246
	Nyala (*Tragelaphus angasii*)	Nyala #1 (S601/20)	n/a	4466	752
Cervidae	Moose (*Alces alces*)	Moose #1 (S612/20)	n/a	19868	693
Giraffidae	Giraffe (*Giraffa sp.)*	Giraffe #1 (S755/20)	n/a	1510009	90756
Camelidae	Camel (*Camelus bactrianus*)	Camel #1 (S747/20)	n/a	2794	43984
	Alpaca (*Vicugna pacos*)	Alpaca #1 (S614/20)	n/a	646367	80572
		Alpaca #2 (S615/20)	n/a	2247	18027
		Alpaca #3 (S758/20)	n/a	4957	37294
Equidae	Horse (*Equus caballus*)	Horse #1 (S583/20)	n/a	8	5
		Horse #2 (S617/20)	n/a	1468	124
		Horse #3 (S633/20)	n/a	26298	10028
		Horse #4 (S649/20)	n/a	298	200
Homonidae	Orangutan (*Pongo sp.*)	Orangutan #1 (S794/20)	n/a	40392	49909

[1]*TMPRSS2* mRNA expression is given as genomic equivalents (GE)/ 1 µg RNA. The table shows mean values for each animal and organ measured as quintuplicates. Threshold: 24.09.
[2]Abbreviations: n/a, no organ sample available; TMPRSS2, transmembrane protease serine subtype 2;

Supplementary Material Table S3. *CTSL* mRNA expression within the upper and lower respiratory tract of mammalian species.

Family	Species	Sample ID	Nasal mucosa	Trachea	Lung
Felidae	Cat (*Felis catus*)	Cat #1 (S528/20)	n/a	2154	282
		Cat #2 (S793/20)	n/a	15721	4827
		Cat #3 (S933/20)	29024	36	51
		Cat #4 (S984/20)	620112	23525	123
		Cat #5 (S148/21)	13992	8737	18485
		Cat #6 (S169/21)	113287	37958	114
	Lion (*Panthera leo*)	Lion #1 (S342/21)	239193	92602	2754
	Cheetah (*Acinonyx jubatus*)	Cheetah #1 (S640/20)	n/a	1222	1065
	Lynx (*Lynx sp.*)	Lynx #1 (S641/20)	n/a	170994	1745
		Lynx #2 (S817/20)	n/a	50263	150
Canidae	Dog (*Canis lupus familiaris*)	Dog #1 (S656/21)	93930	249153	8984
		Dog #2 (S745/21)	51551	66810	7191
		Dog #7 (S773/21)	20353	49694	5777
		Dog #8 (S546/20)	n/a	62359	3535
		Dog #10 (S624/20)	n/a	78608	7292
		Dog #11 (S645/20)	n/a	5507	7525
		Dog #12 (S690/20)	n/a	7772	39001
	Golden Jackal (*Canis aureus*)	Jackal #1 (S542/20)	n/a	1795	6417
Mustelidae	Ferret (*Mustela putorius furo*)	Ferret #1 (V385/20)	383964	198445	34725
		Ferret #3 (V713/20)	n/a	0	100
		Ferret #4 (V749/20)	n/a	121390	30941
		Ferret #7 (V919/20)	n/a	13	742
		Ferret #9 (V920/20)	n/a	0	104
Procyonidae	Raccoon (*Procyon lotor*)	Raccoon #1 (S651/20)	n/a	11760	396

		Raccoon #2 (S652/20)	n/a	10	212
		Raccoon #3 (S656/20)	n/a	2	561
		Raccoon #4 (S697/20)	n/a	299	43
		Raccoon #5 (S775/20)	n/a	116	438
Ailuridae	Red Panda (*Ailurus fulgens*)	Red Panda #1 (S525/20)	n/a	3121	5008
Leporidae	Rabbit (*Oryctolagus cuniculus*)	Rabbit #1 (S715/20)	n/a	113	16
		Rabbit #2 (S946/20)	n/a	16	791
		Rabbit #3 (S1002/20)	n/a	30	15
		Rabbit #4 (S1063/20)	n/a	82	119
Suidae	Pig (*Sus scrofa domesticus*)	Pig #8 (S674/20)	n/a	39100	8930
		Pig #9 (S731/20)	n/a	116039	68207
		Pig #10 (S709/20)	n/a	107680	103
		Pig #11 (S856/20)	n/a	0	2440
	Wild boar (*Sus scrofa*)	Wild boar #1 (S565/20)	n/a	152156	39783
Bovidae	Cattle (*Bos taurus*)	Cattle #2 (S657/20)	n/a	1435	21217
		Cattle #3 (S680/20)	n/a	190435	1115
		Cattle #6 (S765/20)	n/a	125415	26661
		Cattle #7 (S819/20)	n/a	1095	7556
	Sheep (*Ovis aries*)	Sheep #1 (S687/20)	n/a	8733	49
		Sheep #2 (S720/20)	n/a	4269	20
		Sheep #3 (S787/20)	n/a	45793	832
		Sheep #4 (S809/20)	n/a	13032	88
	Goat (*Capra hircus*)	Goat #1 (S602/20)	n/a	3209	0
		Goat #2 (S644/20)	n/a	32	0
		Goat #3 (S722/20)	n/a	5900	0

		Goat #4 (S788/20)	n/a	47949	76945
	Nyala (*Tragelaphus angasii*)	Nyala #1 (S601/20)	n/a	68987	168838
Cervidae	Moose (*Alces alces*)	Moose #1 (S612/20)	n/a	1170	518
Giraffidae	Giraffe (*Giraffa sp.)*	Giraffe #1 (S755/20)	n/a	43968	395
Camelidae	Camel (*Camelus bactrianus*)	Camel #1 (S747/20)	n/a	21414	2
	Alpaca (*Vicugna pacos*)	Alpaca #1 (S614/20)	n/a	36	58
		Alpaca #2 (S615/20)	n/a	418	82
		Alpaca #3 (S758/20)	n/a	67857	0
Equidae	Horse (*Equus caballus*)	Horse #1 (S583/20)	n/a	74	7
		Horse #2 (S617/20)	n/a	321	95
		Horse #3 (S633/20)	n/a	2282	229
		Horse #4 (S649/20)	n/a	527	65
		Horse #5 (S721/20)	n/a	240	233
Homonidae	Orangutan (*Pongo sp.*)	Orangutan #1 (S794/20)	n/a	475	509

[1]*CTSL* mRNA expression is given as genomic equivalents (GE)/ 1 µg RNA. The table shows mean values for each animal and organ measured as quintuplicates. Threshold: primer pair 1: 0.001.
[2]Abbreviations: CTSL, cathepsin L; n/a, no organ sample available;

Supplementary Material Table S4. Sequences of primers used for qPCR and species-specific targets *ACE2, TMPRSS2* and *CTSL*.

GenBank	Primer	ACE2 pan1 forward (5´-3´)																					
	Primer sequence	C	A	A	G	C	A	C	T	T	A	C	A	A	T	T	G	T	T	G	G	A	A
AB211997.1	Cat	.	.	.	.	.	.	.	.	.	.	.	.	.	.	.	.	.	.	.	.	.	.
XM_027054500.1	Cheetah	.	.	.	.	.	.	.	.	.	.	.	.	.	.	.	.	.	.	.	.	.	.
XM_014111329.2	Dog	.	.	.	.	.	.	.	.	.	.	.	.	.	.	.	.	.	.	.	.	.	.
AB208708.1	Ferret	.	.	.	.	.	.	.	.	.	.	.	.	.	.	.	.	.	.	.	.	.	.
-	Golden Jackal	no sequence information available																					
-	Lion	no sequence information available																					
XM_030304979.1	Lynx	.	.	.	.	.	.	.	.	.	.	.	.	.	.	.	.	.	.	.	.	.	.
AB211998.1	Racoon	.	.	.	.	.	.	.	.	.	.	.	.	.	.	.	.	.	.	.	.	.	.
-	Red Panda	no sequence information available																					

GenBank	Primer	ACE2 pan1 reverse (5´-3´)																							
	Primer sequence	T	G	A	G	T	A	A	T	C	A	T	T	A	G	C	A	A	C	A	T	G	G	A	A
AB211997.1	Cat	.	.	.	.	.	.	.	.	.	.	.	.	.	.	.	.	.	.	.	.	.	.	.	.
XM_027054500.1	Cheetah	.	.	.	.	.	.	.	.	.	.	.	.	.	.	.	.	.	.	.	.	.	.	.	.
XM_014111329.2	Dog	.	.	.	.	.	.	.	.	.	.	.	.	.	.	.	.	.	.	.	.	.	.	.	.
AB208708.1	Ferret	.	.	.	.	.	.	.	.	.	G	.	.	.	.	.	.	.	.	.	.	.	.	.	.
-	Golden Jackal	no sequence information available																							
-	Lion	no sequence information available																							
XM_030304979.1	Lynx	.	.	.	.	.	.	.	.	.	.	.	.	.	.	.	.	.	.	.	.	.	.	.	.
AB211998.1	Racoon	C	.	.	.	.	.	.	.	.	.	.	.	.	.	.	.	.	.	.	.	.	.	.	.
-	Red Panda	no sequence information available																							

GenBank	Primer	ACE2 pan2 forward (5´-3´)																			
	Primer sequence	T	G	G	A	G	G	T	G	G	A	T	G	G	T	C	T	T	T	A	A
XM_006212647.3	Alpaca	.	.	.	.	.	.	.	.	.	.	.	.	.	.	.	.	.	.	.	.
XM_010968001.1	Camel	.	.	.	.	.	.	.	.	.	.	.	.	.	.	.	.	.	.	.	.
XM_005228428.4	Cattle	.	.	.	.	.	.	.	.	.	.	.	.	.	.	.	.	.	.	.	.
-	Giraffe	no sequence information available																			
XM_005701072.3	Goat	.	.	.	.	.	.	.	.	.	.	.	.	.	.	.	.	.	.	.	.
XM_001490191.5	Horse	.	.	.	.	.	.	.	.	.	.	.	.	.	.	.	.	.	.	.	.
-	Moose	no sequence information available																			
-	Nyala	no sequence information available																			
NM_001131132.2	Orangutan	.	.	.	.	.	.	.	.	.	.	.	.	.	.	.	.	.	.	.	.
NM_001123070.1	Pig	.	.	.	C	.	.	.	.	.	.	.	.	.	.	.	.	.	.	.	.
XM_002719845.3	Rabbit	.	.	.	.	.	A	.	.	.	.	.	.	.	.	.	.	.	.	.	.
XM_012106267.3	Sheep	.	.	.	.	.	.	.	.	.	.	.	.	.	.	.	.	.	.	.	.

GenBank	Primer	ACE2 pan2 reverse (5´-3´)																							
	Primer sequence	G	A	A	T	T	G	A	T	A	A	A	T	G	G	T	C	C	T	T	G	T	G	T	A
XM_006212647.3	Alpaca	.	.	.	.	.	.	.	.	.	.	.	.	.	.	.	.	.	.	.	.	.	.	.	.
XM_010968001.1	Camel	.	.	.	.	.	.	.	.	.	.	.	.	.	.	.	.	.	.	.	.	.	.	.	.
XM_005228428.4	Cattle	.	.	.	.	.	.	.	.	.	.	.	.	.	.	.	.	.	.	.	.	.	.	.	.
-	Giraffe	no sequence information available																							
XM_005701072.3	Goat	.	.	.	.	.	.	.	.	.	.	.	.	.	.	.	.	.	.	.	.	.	.	.	.
XM_001490191.5	Horse	.	.	.	.	.	.	.	.	.	G	.	.	.	.	.	.	.	.	.	.	.	.	.	.
-	Moose	no sequence information available																							
-	Nyala	no sequence information available																							
NM_001131132.2	Orangutan	.	.	.	.	.	.	G	.	.	.	.	G	.	.	.	.	.	.	.	.				
NM_001123070.1	Pig	.	.	.	C	.	.	.	.	.	.	.	.	.	.	.	.	.	.	.	.	.	.	.	.
XM_002719845.3	Rabbit	.	.	.	.	.	A	.	.	.	.	.	.	T	.	.	.	.	.	G	.	.	.	.	.

XM_012106267.3	Sheep	.	.	.	.	.	.	.	.	.	.	.	.	.	.	.	.	.	.	.	.	.	.	.	.

GenBank	Primer	TMPRSS2 pan forward (5´-3´)																	
	Primer sequence	T	G	G	A	G	G	T	C	C	Y	C	T	G	G	T	C	A	C
XM_031684299.1	Alpaca	.	.	.	.	.	.	.	.	.	T	.	.	.	.	.	.	.	.
XM_010994865.2	Camel	C	.	.	.	.	.	.	.	.	T	.	.	.	.	.	.	.	.
XM_023238711.1	Cat	.	.	.	.	.	.	C	.	.	C	.	.	.	.	.	.	.	.
NM_001081585.1	Cattle	.	.	.	.	.	.	.	.	.	T	.	.	.	.	.	.	.	.
XM_027041576.1	Cheetah	.	.	.	.	.	.	C	.	.	C	.	.	.	.	.	.	.	.
LC219373.1	Dog	C	.	.	.	.	.	.	.	.	C	.	.	.	.	.	.	.	.
NM_001386127.1	Ferret	C	.	.	.	.	.	.	.	.	C	.	.	.	.	.	.	.	.
-	Giraffe	no sequence information available																	
XM_005675629.3	Goat	.	.	.	.	.	.	.	.	.	.	.	.	.	.	.	.	.	.
-	Golden Jackal	no sequence information available																	
XM_023630207.1	Horse	.	.	.	.	.	.	.	.	.	T	.	.	.	.	.	.	.	.
XM_042956092.1	Lion	.	.	.	.	.	.	C	.	.	C	.	.	.	.	.	.	.	.
XM_030330005.1	Lynx	.	.	.	.	.	.	C	.	.	C	.	.	.	.	.	.	.	.
-	Moose	no sequence information available																	
-	Nyala	no sequence information available																	
XM_024239539.1	Orangutan	.	.	.	.	.	.	G	.	.	T	.	.	.	.	.	.	.	.
NM_001386131.1	Pig	C	.	.	.	.	.	.	.	.	T	.	.	.	.	.	.	.	.
NM_001386128.1	Rabbit	C	.	.	C	.	.	G	.	.	T	.	.	.	.	.	.	.	.
-	Racoon	no sequence information available																	
-	Red Panda	no sequence information available																	
XM_027960704.2	Sheep	.	.	.	.	.	.	.	.	.	T	.	.	.	.	.	.	.	.

GenBank	Primer	TMPRSS2 pan reverse (5´-3´)																			
	Primer sequence	A	T	T	T	G	T	C	G	A	T	A	R	A	T	C	C	A	G	T	C
XM_031684299.1	Alpaca	.	.	.	.	.	.	.	.	.	.	.	A	.	.	.	.	.	.	.	.
XM_010994865.2	Camel	.	.	.	.	.	.	.	.	.	.	.	A	.	.	.	.	.	.	.	.
XM_023238711.1	Cat	.	.	.	.	.	.	.	.	.	.	.	A	.	.	.	.	.	.	.	.
NM_001081585.1	Cattle	.	.	.	.	.	.	T	.	.	.	.	G	.	.	.	.	.	.	.	.
XM_027041576.1	Cheetah	.	.	.	.	.	.	.	.	G	.	.	A	.	.	.	.	.	.	.	.
LC219373.1	Dog	.	.	.	.	.	C	.	.	.	.	.	A	.	.	.	.	.	.	.	.
NM_001386127.1	Ferret	.	.	.	.	.	.	.	.	G	.	.	A	.	.	.	.	.	.	.	.
-	Giraffe	no sequence information available																			
XM_005675629.3	Goat	.	.	.	.	.	.	.	.	.	.	.	G	.	.	.	.	.	.	.	.
-	Golden Jackal	no sequence information available																			
XM_023630207.1	Horse	.	.	C	.	C	.	T	.	G	.	.	G	.	.	.	.	.	.	.	.
XM_042956092.1	Lion	.	.	.	.	.	.	.	.	.	.	.	A	.	.	.	.	.	.	.	.
XM_030330005.1	Lynx	.	.	.	.	.	.	.	.	.	.	.	A	.	.	.	.	.	.	.	.
-	Moose	no sequence information available																			
-	Nyala	no sequence information available																			
XM_024239539.1	Orangutan	.	.	.	.	.	.	.	.	.	.	.	A	.	.	.	.	.	.	.	.
NM_001386131.1	Pig	.	.	.	.	.	.	.	.	.	.	.	A	.	.	.	.	.	.	.	.
NM_001386128.1	Rabbit	.	.	C	.	.	C	T	.	G	.	.	G	.	.	.	.	.	.	.	.
-	Racoon	no sequence information available																			
-	Red Panda	no sequence information available																			
XM_027960704.2	Sheep	.	.	.	.	.	.	.	.	.	.	.	G	.	.	.	.	.	.	.	.

GenBank	Primer	CTSL pan forward (5´-3´)																								
	Primer sequence	A	T	G	A	A	Y	G	C	Y	T	T	Y	G	G	H	G	A	C	A	T	G	A	C	C	A
XM_006209235.3	Alpaca	.	.	.	.	.	T	.	.	C	.	.	T	.	.	T	.	.	.	.	.	.	.	.	.	.
XM_031447264.1	Camel	.	.	.	.	.	T	.	.	C	.	.	T	.	.	T	.	.	.	.	.	.	.	.	.	.
XM_011288293.3	Cat	.	.	.	.	.	T	.	.	T	.	.	T	.	.	T	.	.	.	.	.	.	.	.	.	.
NM_174032.2	Cattle	.	.	.	.	.	T	.	.	C	.	.	T	.	.	T	.	.	.	.	.	.	.	.	.	.
-	Cheetah	no sequence information available																								
XM_038654910.1	Dog	.	.	.	.	.	T	.	.	C	.	.	T	.	.	T	.	.	.	.	.	.	.	.	.	.
XM_004777423.3	Ferret	.	.	.	.	.	T	.	.	C	.	.	C	.	.	T	.	.	.	.	.	.	.	.	.	.
-	Giraffe	no sequence information available																								
NM_001314266.1	Goat	.	.	.	.	.	T	.	.	C	.	.	T	.	.	T	.	.	.	.	.	.	.	.	.	.
-	Golden Jackal	no sequence information available																								
XM_023627064.1	Horse	.	.	.	.	.	T	.	.	C	.	.	T	.	.	T	.	.	.	.	.	.	.	.	.	.
XM_042912503.1	Lion	.	.	.	.	.	T	.	G	C	.	.	T	.	.	T	.	.	.	.	.	.	.	.	.	.
XM_030293577.1	Lynx	.	.	.	.	.	T	.	.	C	.	.	T	.	.	T	.	.	.	.	.	.	.	.	.	.
-	Moose	no sequence information available																								
-	Nyala	no sequence information available																								
XM_002820009.3	Orangutan	.	.	.	.	.	T	.	.	T	.	.	T	.	.	T	.	.	.	.	.	.	.	.	.	.
XM_003130633.6	Pig	.	.	.	.	.	T	.	.	C	.	.	T	.	.	T	.	.	.	.	.	.	.	.	.	.
XM_002708253.3	Rabbit	.	.	.	.	.	C	.	.	C	.	A	C	.	.	C	.	.	.	.	.	.	.	.	.	.
-	Racoon	no sequence information available																								
-	Red Panda	no sequence information available																								
XM_004004081.4	Sheep	.	.	.	.	.	T	.	.	C	.	.	T	.	.	T	.	.	.	.	.	.	.	.	.	.

GenBank	Primer														CTSL pan reverse (5´-3´)										
	Primer sequence	C	C	A	G	T	K	G	C	A	C	T	A	A	A	M	G	C	C	C	A	R	C	A	A
XM_006209235.3	Alpaca	.	.	.	.	.	G	.	.	.	.	.	.	.	.	G	.	.	.	.	.	A	.	.	.
XM_031447264.1	Camel	.	.	.	.	.	G	.	.	.	.	.	G	.	.	A	.	.	.	.	.	A	.	.	.
XM_011288293.3	Cat	.	.	.	.	.	T	.	.	.	.	.	.	.	.	A	.	.	.	.	.	A	.	.	.
NM_174032.2	Cattle	.	.	.	.	.	G	.	.	.	.	.	.	.	.	C	.	.	.	.	.	A	.	.	.
-	Cheetah	no sequence information available																							
XM_038654910.1	Dog	.	.	.	.	.	T	.	.	.	.	.	.	.	.	A	.	.	.	.	.	A	.	.	.
XM_004777423.3	Ferret	.	.	.	.	.	T	.	.	.	.	.	.	.	.	A	.	.	.	.	.	A	.	.	.
-	Giraffe	no sequence information available																							
NM_001314266.1	Goat	.	.	.	.	.	G	.	.	.	.	.	.	.	.	C	.	.	.	.	.	A	.	.	.
-	Golden Jackal	no sequence information available																							
XM_023627064.1	Horse	.	.	.	.	.	C	.	.	.	.	.	.	.	.	A	.	.	.	.	.	A	.	.	.
XM_042912503.1	Lion	.	.	.	.	.	T	.	.	.	.	.	.	.	.	A	.	.	.	.	.	A	.	.	.
XM_030293577.1	Lynx	.	.	.	.	.	T	.	.	.	.	.	.	.	.	A	.	.	.	.	.	A	.	.	.
-	Moose	no sequence information available																							
-	Nyala	no sequence information available																							
XM_002820009.3	Orangutan	.	.	.	.	.	C	.	.	.	.	.	.	.	.	A	.	.	.	.	.	A	.	.	.
XM_003130633.6	Pig	.	.	G	.	.	G	.	.	.	.	.	.	.	.	A	.	.	.	.	.	A	.	.	.
XM_002708253.3	Rabbit	.	.	.	.	.	G	.	.	.	.	.	G	.	.	A	.	.	.	.	.	A	.	.	.
-	Racoon	no sequence information available																							
-	Red Panda	no sequence information available																							
XM_004004081.4	Sheep	.	.	.	.	.	G	.	.	.	.	.	.	.	.	C	.	.	.	.	.	A	.	.	.

[1]Abbreviations: ACE2, angiotensin-converting enzyme 2; CTSL, cathepsin L; TMPRSS2, transmembrane protease serine subtype 2;

Supplementary Material Table S5. Description of evaluation criteria of cytopathic features in culture systems used in the present study.

Sample	Cytopathic feature	Type of evaluation
NME	Ciliated epithelial cells	quantitative, (%) in relation to total number of epithelial cells
	Cytoplasmic vacuolization in the epithelium	
	Nuclear degeneration in the epithelium	
	Cytoplasmic vacuolization in the submucosal glands	quantitative, number of cells with changes in relation to total number of submucosal glands
	Nuclear degeneration in the submucosal glands	
	Cytoplasmic vacuolization in the connective tissue	quantitative, number of cells with changes in relation to total area of connective tissue quantified in HPF
	Nuclear degeneration in the connective tissue	
	Vascular edema	semiquantitative, scoring 1-4 (1= none, 2= mild, 3= moderate, 4= severe)
ALI	Total cell count (per HPF)	quantitative
	Ciliated epithelial cells (per HPF)	quantitative, (%) in relation to total cell count
	Cytoplasmic vacuolization (per HPF)	quantitative, (%) in relation to total cell count
	Nuclear degeneration (per HPF)	quantitative, (%) in relation to total cell count
PCLS	Ciliated epithelial cells	quantitative, (%) in relation to total number of bronchial epithelial cells
	Cytoplasmic vacuolization in the bronchial epithelium	
	Nuclear degeneration in the bronchial epithelium	
	Cytoplasmic vacuolization in the peribronchial glands	quantitative, number of cells with changes in relation to total number of peribronchial glands
	Nuclear degeneration in the peribronchial glands	
	Cytoplasmic vacuolization in the peribronchial connective tissue	quantitative, number of cells with changes in relation to total area of connective tissue quantified in HPF
	Nuclear degeneration in the peribronchial connective tissue	
	Cytoplasmic vacuolization in the alveolar interstitium	quantitative, number of cells with changes in five HPF
	Nuclear degeneration in the alveolar interstitium	
	Cells in the alveolar lumen	
	Vascular edema	semiquantitative, scoring 1-4 (1= none, 2= mild, 3= moderate, 4= severe)

[1]Abbreviations: ALI, air-liquid interface cultures; HPF, high power field (400x magnification); NME, nasal mucosa explants; PCLS, precision-cut lung slices;

Supplementary Material Table S6. Primary antibodies used for immunofluorescence (IF) and immunohistochemistry (IHC).

**Antibody**	**Specificity**	**Target**	**Dilution**	**Positive control (from the investigated species)**
SARS-CoV-2 NP (SinoBiological - 40143); IF	Mouse, Monoclonal, clone #05	SARS-CoV-2 nucleoprotein (NP)	1:500	Hamster lung, experimentally infected with SARS-CoV-2 [50], 1 dpi
Caspase-3 (Promega - G748A); IHC	Rabbit, Polyclonal	apoptotic cells	1:200	lymph node
CD204 (Abnova Corporation - MAB1710); IHC	Mouse, Monoclonal clone SRA-E5	macrophages	1:500	lymph node
α-tubulin (Sigma-Aldrich - T6793); IHC	Mouse, Monoclonal, clone 6-11B-1	ciliated cells	1:100	brain
Ki67 (DakoCytomation - GA62661-2); IHC	Mouse, Monoclonal, clone MIB-1	proliferating cells	1:100	intestine

[1]Abbreviations: CD204, cluster of differentiation 204; dpi, days post infection; SARS-CoV-2, severe acute respiratory syndrome coronavirus 2;

Supplementary Material Table S7. Light microscopic evaluation of hematoxylin and eosin stained culture systems. Pairwise comparisons of controls and SARS-CoV-2 infected samples, p-values significant at ≤0,05 obtained by Mann-Whitney-U tests.

days p.i.	Group	Median	p-value
**Total cell count**			
1	ALI cow (control)	231,000	0,114
1	ALI cow (SARS-CoV-2)	200,000	
5	ALI cow (control)	196,500	**0,007**
5	ALI cow (SARS-CoV-2)	167,000	
**Ciliated epithelial cells**			
1	NME dog (control)	0,000	0,743
1	NME dog (SARS-CoV-2)	0,000	
2	NME dog (control)	0,646	1,000
2	NME dog (SARS-CoV-2)	0,976	
3	NME dog (control)	0,000	0,748
3	NME dog (SARS-CoV-2)	0,000	
4	NME dog (control)	0,000	0,750
4	NME dog (SARS-CoV-2)	0,000	
1	ALI cow (control)	14,894	0,756
1	ALI cow (SARS-CoV-2)	12,097	
5	ALI cow (control)	12,571	**0,000**
5	ALI cow (SARS-CoV-2)	23,940	
1	PCLS dog (control)	1,563*	0,400
1	PCLS dog (SARS-CoV-2)	2,145	
2	PCLS dog (control)	n.e.	n.e.
2	PCLS dog (SARS-CoV-2)	0,000	
3	PCLS dog (control)	n.e.	n.e.
3	PCLS dog (SARS-CoV-2)	n.e.	
4	PCLS dog (control)	0,000*	1,000
4	PCLS dog (SARS-CoV-2)	0,000*	
1	PCLS hamster (control)	67,368	0,400
1	PCLS hamster (SARS-CoV-2)	75,510	
2	PCLS hamster (control)	45,983	1,000
2	PCLS hamster (SARS-CoV-2)	32,268	
3	PCLS hamster (control)	54,151	0,667
3	PCLS hamster (SARS-CoV-2)	81,928*	
4	PCLS hamster (control)	81,035	1,000
4	PCLS hamster (SARS-CoV-2)	78,723	
1	PCLS human (control)	45,674	0,333
1	PCLS human (SARS-CoV-2)	73,462	
2	PCLS human (control)	74,324	1,000
2	PCLS human (SARS-CoV-2)	55,131	
3	PCLS human (control)	62,157	0,667

3	PCLS human (SARS-CoV-2)	20,635*	
4	PCLS human (control)	70,427	0,667
4	PCLS human (SARS-CoV-2)	78,782	
**Cytoplasmic vacuolisation (epithelium)**			
1	NME dog (control)	1,792	0,481
1	NME dog (SARS-CoV-2)	2,788	
2	NME dog (control)	2,865	0,700
2	NME dog (SARS-CoV-2)	4,434	
3	NME dog (control)	6,122	0,332
3	NME dog (SARS-CoV-2)	3,650	
4	NME dog (control)	4,221	0,617
4	NME dog (SARS-CoV-2)	3,509	
1	ALI cow (control)	28,804	**0,017**
1	ALI cow (SARS-CoV-2)	21,223	
5	ALI cow (control)	25,210	0,417
5	ALI cow (SARS-CoV-2)	27,439	
1	PCLS dog (control)	1,563*	0,800
1	PCLS dog (SARS-CoV-2)	2,610	
2	PCLS dog (control)	n.e.	n.e.
2	PCLS dog (SARS-CoV-2)	0,000	
3	PCLS dog (control)	n.e.	n.e.
3	PCLS dog (SARS-CoV-2)	0,000	
4	PCLS dog (control)	0,000	1,000
4	PCLS dog (SARS-CoV-2)	5,660	
1	PCLS hamster (control)	1,630	1,000
1	PCLS hamster (SARS-CoV-2)	1,415	
2	PCLS hamster (control)	1,548	1,000
2	PCLS hamster (SARS-CoV-2)	1,635	
3	PCLS hamster (control)	1,409	1,000
3	PCLS hamster (SARS-CoV-2)	1,205	
4	PCLS hamster (control)	1,724	0,383
4	PCLS hamster (SARS-CoV-2)	1,418	
1	PCLS human (control)	2,150	0,333
1	PCLS human (SARS-CoV-2)	3,932	
2	PCLS human (control)	2,783	1,000
2	PCLS human (SARS-CoV-2)	2,643	
3	PCLS human (control)	3,391	1,000
3	PCLS human (SARS-CoV-2)	2,381*	
4	PCLS human (control)	2,699	1,000
4	PCLS human (SARS-CoV-2)	1,263	
**Cytoplasmic vacuolisation (submucosal/peribronchial glands)**			
1	NME dog (control)	0,349	0,739
1	NME dog (SARS-CoV-2)	0,275	
2	NME dog (control)	0,412	1,000
2	NME dog (SARS-CoV-2)	0,546	
3	NME dog (control)	0,400	0,519

3	NME dog (SARS-CoV-2)	0,286	
4	NME dog (control)	0,231	0,842
4	NME dog (SARS-CoV-2)	0,250	
1	PCLS dog (control)	0,250*	0,400
1	PCLS dog (SARS-CoV-2)	0,528	
2	PCLS dog (control)	0,000*	0,800
2	PCLS dog (SARS-CoV-2)	0,071	
3	PCLS dog (control)	0,083*	0,500
3	PCLS dog (SARS-CoV-2)	0,000	
4	PCLS dog (control)	0,000*	0,800
4	PCLS dog (SARS-CoV-2)	0,056	
1	PCLS hamster (control)	0,000*	1,000
1	PCLS hamster (SARS-CoV-2)	0,000*	
2	PCLS hamster (control)	n.e.	n.e.
2	PCLS hamster (SARS-CoV-2)	n.e.	
3	PCLS hamster (control)	n.e.	n.e.
3	PCLS hamster (SARS-CoV-2)	n.e.	
4	PCLS hamster (control)	0,000*	1,000
4	PCLS hamster (SARS-CoV-2)	0,000*	
1	PCLS human (control)	0,000*	1,000
1	PCLS human (SARS-CoV-2)	0,000*	
2	PCLS human (control)	2,000*	n.a
2	PCLS human (SARS-CoV-2)	n.e.	
3	PCLS human (control)	1,000*	n.a
3	PCLS human (SARS-CoV-2)	n.e.	
4	PCLS human (control)	n.e.	n.a
4	PCLS human (SARS-CoV-2)	n.e.	
**Cytoplasmic vacuolisation (connective tissue)**			
1	NME dog (control)	0,000	1,000
1	NME dog (SARS-CoV-2)	0,000	
2	NME dog (control)	0,000	1,000
2	NME dog (SARS-CoV-2)	0,000	
3	NME dog (control)	0,000	1,000
3	NME dog (SARS-CoV-2)	0,000	
4	NME dog (control)	0,000	1,000
4	NME dog (SARS-CoV-2)	0,000	
1	PCLS dog (control)	0,000*	1,000
1	PCLS dog (SARS-CoV-2)	0,000	
2	PCLS dog (control)	0,000*	1,000
2	PCLS dog (SARS-CoV-2)	0,000	
3	PCLS dog (control)	0,000*	1,000
3	PCLS dog (SARS-CoV-2)	0,000	
4	PCLS dog (control)	0,000*	1,000
4	PCLS dog (SARS-CoV-2)	0,000	
1	PCLS hamster (control)	0,000	1,000
1	PCLS hamster (SARS-CoV-2)	0,000	

2	PCLS hamster (control)	0,250	0,667
2	PCLS hamster (SARS-CoV-2)	0,000	
3	PCLS hamster (control)	0,000	1,000
3	PCLS hamster (SARS-CoV-2)	0,000*	
4	PCLS hamster (control)	0,000	0,683
4	PCLS hamster (SARS-CoV-2)	0,000	
1	PCLS human (control)	0,000	1,000
1	PCLS human (SARS-CoV-2)	0,000	
2	PCLS human (control)	0,000	1,000
2	PCLS human (SARS-CoV-2)	0,000	
3	PCLS human (control)	0,000	1,000
3	PCLS human (SARS-CoV-2)	0,000*	
4	PCLS human (control)	0,000	1,000
4	PCLS human (SARS-CoV-2)	0,000	
**Cytoplasmic vacuolisation (alveolar interstitium)**			
1	PCLS dog (control)	0,000	0,974
1	PCLS dog (SARS-CoV-2)	0,000	
2	PCLS dog (control)	0,000	0,371
2	PCLS dog (SARS-CoV-2)	0,000	
3	PCLS dog (control)	0,000	0,530
3	PCLS dog (SARS-CoV-2)	0,000	
4	PCLS dog (control)	0,000	0,717
4	PCLS dog (SARS-CoV-2)	0,000	
1	PCLS hamster (control)	1,000	0,744
1	PCLS hamster (SARS-CoV-2)	1,000	
2	PCLS hamster (control)	0,000	0,056
2	PCLS hamster (SARS-CoV-2)	0,000	
3	PCLS hamster (control)	0,000	0,512
3	PCLS hamster (SARS-CoV-2)	0,000	
4	PCLS hamster (control)	0,000	0,769
4	PCLS hamster (SARS-CoV-2)	0,000	
1	PCLS human (control)	0,000	0,481
1	PCLS human (SARS-CoV-2)	0,000	
2	PCLS human (control)	0,000	0,739
2	PCLS human (SARS-CoV-2)	0,000	
3	PCLS human (control)	0,000	0,481
3	PCLS human (SARS-CoV-2)	0,000	
4	PCLS human (control)	0,000	0,481
4	PCLS human (SARS-CoV-2)	0,000	
**Nuclear degeneration (epithelium)**			
1	NME dog (control)	0,785	0,200
1	NME dog (SARS-CoV-2)	1,604	
2	NME dog (control)	1,302	0,700
2	NME dog (SARS-CoV-2)	3,179	
3	NME dog (control)	1,914	0,699
3	NME dog (SARS-CoV-2)	3,398	

4	NME dog (control)	2,625	0,820
4	NME dog (SARS-CoV-2)	3,680	
1	ALI cow (control)	6,957	0,349
1	ALI cow (SARS-CoV-2)	6,299	
5	ALI cow (control)	6,254	0,830
5	ALI cow (SARS-CoV-2)	5,955	
1	PCLS dog (control)	1,563*	1,000
1	PCLS dog (SARS-CoV-2)	1,453	
2	PCLS dog (control)	n.e.	n.e.
2	PCLS dog (SARS-CoV-2)	0,909	
3	PCLS dog (control)	n.e.	n.e.
3	PCLS dog (SARS-CoV-2)	n.e.	
4	PCLS dog (control)	0,000*	1,000
4	PCLS dog (SARS-CoV-2)	3,774*	
1	PCLS hamster (control)	2,105	0,400
1	PCLS hamster (SARS-CoV-2)	4,082	
2	PCLS hamster (control)	5,380	0,333
2	PCLS hamster (SARS-CoV-2)	9,056	
3	PCLS hamster (control)	9,674	0,667
3	PCLS hamster (SARS-CoV-2)	3,615*	
4	PCLS hamster (control)	4,310	0,833
4	PCLS hamster (SARS-CoV-2)	4,478	
1	PCLS human (control)	1,465	0,333
1	PCLS human (SARS-CoV-2)	3,932	
2	PCLS human (control)	2,285	0,333
2	PCLS human (SARS-CoV-2)	0,983	
3	PCLS human (control)	1,215	0,667
3	PCLS human (SARS-CoV-2)	3,175*	
4	PCLS human (control)	2,081	0,333
4	PCLS human (SARS-CoV-2)	0,936	
**Nuclear degeneration (submucosal/peribronchial glands)**			
1	NME dog (control)	0,354	0,105
1	NME dog (SARS-CoV-2)	0,145	
2	NME dog (control)	0,375	0,700
2	NME dog (SARS-CoV-2)	0,455	
3	NME dog (control)	0,429	0,949
3	NME dog (SARS-CoV-2)	0,417	
4	NME dog (control)	0,444	0,445
4	NME dog (SARS-CoV-2)	0,428	
1	PCLS dog (control)	0,500*	0,800
1	PCLS dog (SARS-CoV-2)	0,330	
2	PCLS dog (control)	2,000*	0,400
2	PCLS dog (SARS-CoV-2)	0,615	
3	PCLS dog (control)	0,583*	0,500
3	PCLS dog (SARS-CoV-2)	0,800	
4	PCLS dog (control)	1,000*	0,400

4	PCLS dog (SARS-CoV-2)	0,528	
1	PCLS hamster (control)	1,000*	1,000
1	PCLS hamster (SARS-CoV-2)	2,000*	
2	PCLS hamster (control)	n.e.	n.e.
2	PCLS hamster (SARS-CoV-2)	n.e.	
3	PCLS hamster (control)	n.e.	n.e.
3	PCLS hamster (SARS-CoV-2)	n.e.	
4	PCLS hamster (control)	2,000*	1,000
4	PCLS hamster (SARS-CoV-2)	3,000*	
1	PCLS human (control)	1,000*	1,000
1	PCLS human (SARS-CoV-2)	0,000*	
2	PCLS human (control)	1,000*	n.e.
2	PCLS human (SARS-CoV-2)	n.e.	
3	PCLS human (control)	0,000*	n.e.
3	PCLS human (SARS-CoV-2)	n.e.	
4	PCLS human (control)	n.e.	n.e.
4	PCLS human (SARS-CoV-2)	n.e.	
**Nuclear degeneration (connective tissue)**			
1	NME dog (control)	0,888	0,436
1	NME dog (SARS-CoV-2)	0,976	
2	NME dog (control)	2,444	1,000
2	NME dog (SARS-CoV-2)	2,667	
3	NME dog (control)	1,056	0,519
3	NME dog (SARS-CoV-2)	0,889	
4	NME dog (control)	0,831	0,407
4	NME dog (SARS-CoV-2)	1,557	
1	PCLS dog (control)	1,500*	0,800
1	PCLS dog (SARS-CoV-2)	1,196	
2	PCLS dog (control)	2,000*	0,800
2	PCLS dog (SARS-CoV-2)	3,150	
3	PCLS dog (control)	0,909*	0,500
3	PCLS dog (SARS-CoV-2)	5,163	
4	PCLS dog (control)	4,000*	0,800
4	PCLS dog (SARS-CoV-2)	4,171	
1	PCLS hamster (control)	2,000	0,700
1	PCLS hamster (SARS-CoV-2)	7,333	
2	PCLS hamster (control)	9,083	0,667
2	PCLS hamster (SARS-CoV-2)	4,500	
3	PCLS hamster (control)	4,000	0,667
3	PCLS hamster (SARS-CoV-2)	7,333*	
4	PCLS hamster (control)	3,000	0,683
4	PCLS hamster (SARS-CoV-2)	3,000	
1	PCLS human (control)	0,900	1,000
1	PCLS human (SARS-CoV-2)	1,250	
2	PCLS human (control)	1,083	0,333
2	PCLS human (SARS-CoV-2)	0,750	

3	PCLS human (control)	0,543	0,667
3	PCLS human (SARS-CoV-2)	0,000*	
4	PCLS human (control)	1,550	0,333
4	PCLS human (SARS-CoV-2)	1,129	
**Nuclear degeneration (alveolar interstitium)**			
1	PCLS dog (control)	0,000	0,071
1	PCLS dog (SARS-CoV-2)	1,000	
2	PCLS dog (control)	1,000	0,169
2	PCLS dog (SARS-CoV-2)	0,000	
3	PCLS dog (control)	0,000	0,097
3	PCLS dog (SARS-CoV-2)	0,500	
4	PCLS dog (control)	2,000	**0,000**
4	PCLS dog (SARS-CoV-2)	0,000	
1	PCLS hamster (control)	3,000	0,775
1	PCLS hamster (SARS-CoV-2)	2,000	
2	PCLS hamster (control)	5,000	0,436
2	PCLS hamster (SARS-CoV-2)	6,000	
3	PCLS hamster (control)	7,000	0,161
3	PCLS hamster (SARS-CoV-2)	2,000	
4	PCLS hamster (control)	6,500	0,663
4	PCLS hamster (SARS-CoV-2)	5,000	
1	PCLS human (control)	1,000	0,529
1	PCLS human (SARS-CoV-2)	1,000	
2	PCLS human (control)	1,000	0,190
2	PCLS human (SARS-CoV-2)	0,000	
3	PCLS human (control)	1,000	0,353
3	PCLS human (SARS-CoV-2)	0,500	
4	PCLS human (control)	0,500	0,912
4	PCLS human (SARS-CoV-2)	0,000	
**Cells in alveolar lumen**			
1	PCLS dog (control)	1,000	0,575
1	PCLS dog (SARS-CoV-2)	2,000	
2	PCLS dog (control)	4,000	**0,006**
2	PCLS dog (SARS-CoV-2)	1,000	
3	PCLS dog (control)	0,000	0,083
3	PCLS dog (SARS-CoV-2)	2,000	
4	PCLS dog (control)	2,000	0,192
4	PCLS dog (SARS-CoV-2)	1,000	
1	PCLS hamster (control)	1,000	0,116
1	PCLS hamster (SARS-CoV-2)	0,000	
2	PCLS hamster (control)	0,000	0,202
2	PCLS hamster (SARS-CoV-2)	0,000	
3	PCLS hamster (control)	0,000	0,325
3	PCLS hamster (SARS-CoV-2)	0,000	
4	PCLS hamster (control)	0,000	0,989
4	PCLS hamster (SARS-CoV-2)	0,000	

1	PCLS human (control)	1,000	0,579
1	PCLS human (SARS-CoV-2)	0,500	
2	PCLS human (control)	0,000	0,247
2	PCLS human (SARS-CoV-2)	0,500	
3	PCLS human (control)	0,000	0,436
3	PCLS human (SARS-CoV-2)	0,000	
4	PCLS human (control)	0,500	0,912
4	PCLS human (SARS-CoV-2)	1,000	
**Vascular edema**			
1	NME dog (control)	1,000	0,143
1	NME dog (SARS-CoV-2)	1,000	
2	NME dog (control)	1,000	1,000
2	NME dog (SARS-CoV-2)	1,000	
3	NME dog (control)	1,000	0,654
3	NME dog (SARS-CoV-2)	1,000	
4	NME dog (control)	1,000	0,240
4	NME dog (SARS-CoV-2)	2,000	
1	PCLS dog (control)	4,000*	1,000
1	PCLS dog (SARS-CoV-2)	4,000	
2	PCLS dog (control)	4,000*	1,000
2	PCLS dog (SARS-CoV-2)	4,000	
3	PCLS dog (control)	4,000*	1,000
3	PCLS dog (SARS-CoV-2)	4,000	
4	PCLS dog (control)	1,000*	0,500
4	PCLS dog (SARS-CoV-2)	4,000	
1	PCLS hamster (control)	4,000	1,000
1	PCLS hamster (SARS-CoV-2)	4,000	
2	PCLS hamster (control)	4,000	1,000
2	PCLS hamster (SARS-CoV-2)	4,000*	
3	PCLS hamster (control)	4,000	1,000
3	PCLS hamster (SARS-CoV-2)	4,000	
4	PCLS hamster (control)	4,000	1,000
4	PCLS hamster (SARS-CoV-2)	4,000	
1	PCLS human (control)	3,000*	0,667
1	PCLS human (SARS-CoV-2)	4,000	
2	PCLS human (control)	4,000	1,000
2	PCLS human (SARS-CoV-2)	4,000	
3	PCLS human (control)	4,000	1,000
3	PCLS human (SARS-CoV-2)	4,000	
4	PCLS human (control)	4,000*	1,000
4	PCLS human (SARS-CoV-2)	4,000	

[1]* this value is based on one cell culture

[2]The bold font indicates significance.

[3]Abbreviations: ALI, air-liquid interface cultures; dpi, days post infection; n.e., not evaluable, due to lack of target structure; NME, nasal mucosa explants; PCLS, precision-cut lung slices; SARS-CoV-2, severe acute respiratory syndrome coronavirus 2;

Supplementary Material Table S8. Light microscopic evaluation of immunohistochemically stained culture systems. Pairwise comparisons of controls and SARS-CoV-2 infected samples, p-values significant at ≤0,05 obtained by Mann-Whitney-U tests.

days p.i.	Group	Median	p-value
**α-tubulin positive cells**			
1	NME dog (control)	0,000	0,842
1	NME dog (SARS-CoV-2)	0,000	
2	NME dog (control)	0,182	0,700
2	NME dog (SARS-CoV-2)	0,625	
3	NME dog (control)	0,000	1,000
3	NME dog (SARS-CoV-2)	0,000	
4	NME dog (control)	0,000	0,842
4	NME dog (SARS-CoV-2)	0,000	
1	ALI cow (control)	14,890	0,756
1	ALI cow (SARS-CoV-2)	12,100	
5	ALI cow (control)	12,570	**0,000**
5	ALI cow (SARS-CoV-2)	23,610	
1	PCLS dog (control)	0,3333*	0,400
1	PCLS dog (SARS-CoV-2)	0,875	
2	PCLS dog (control)	n.e.	n.e.
2	PCLS dog (SARS-CoV-2)	0,000	
3	PCLS dog (control)	0*	1,000
3	PCLS dog (SARS-CoV-2)	0,000	
4	PCLS dog (control)	n.e.	n.e.
4	PCLS dog (SARS-CoV-2)	0,000	
1	PCLS hamster (control)	31,000	0,400
1	PCLS hamster (SARS-CoV-2)	40,000	
2	PCLS hamster (control)	14,738	1,000
2	PCLS hamster (SARS-CoV-2)	15,417	
3	PCLS hamster (control)	13,733	1,000
3	PCLS hamster (SARS-CoV-2)	16,667	
4	PCLS hamster (control)	29,417	0,933
4	PCLS hamster (SARS-CoV-2)	28,600	
1	PCLS human (control)	27,551	1,000
1	PCLS human (SARS-CoV-2)	28,333	
2	PCLS human (control)	25,558	0,667
2	PCLS human (SARS-CoV-2)	16,857	
3	PCLS human (control)	34,717	0,667
3	PCLS human (SARS-CoV-2)	7,600*	
4	PCLS human (control)	22,750	0,333
4	PCLS human (SARS-CoV-2)	32,182	
**Caspase-3 positive cells**			
1	NME dog (control)	9,500	0,739

1	NME dog (SARS-CoV-2)	8,500	
2	NME dog (control)	15,000	0,700
2	NME dog (SARS-CoV-2)	13,000	
3	NME dog (control)	5,000	**0,019**
3	NME dog (SARS-CoV-2)	9,000	
4	NME dog (control)	6,500	0,261
4	NME dog (SARS-CoV-2)	8,000	
1	ALI cow (control)	0,010	0,756
1	ALI cow (SARS-CoV-2)	0,020	
5	ALI cow (control)	1,820	0,618
5	ALI cow (SARS-CoV-2)	2,096	
1	PCLS dog (control)	15*	0,800
1	PCLS dog (SARS-CoV-2)	13,500	
2	PCLS dog (control)	8*	0,400
2	PCLS dog (SARS-CoV-2)	13,000	
3	PCLS dog (control)	14*	0,800
3	PCLS dog (SARS-CoV-2)	9,500	
4	PCLS dog (control)	17*	1,000
4	PCLS dog (SARS-CoV-2)	15,500	
1	PCLS hamster (control)	6,000	1,000
1	PCLS hamster (SARS-CoV-2)	6,000	
2	PCLS hamster (control)	7,000	0,700
2	PCLS hamster (SARS-CoV-2)	10,000	
3	PCLS hamster (control)	11,000	0,400
3	PCLS hamster (SARS-CoV-2)	10,000	
4	PCLS hamster (control)	5,500	0,607
4	PCLS hamster (SARS-CoV-2)	7,000	
1	PCLS human (control)	4,500	0,667
1	PCLS human (SARS-CoV-2)	2,000	
2	PCLS human (control)	6,500	0,333
2	PCLS human (SARS-CoV-2)	2,500	
3	PCLS human (control)	9,000	0,667
3	PCLS human (SARS-CoV-2)	7,000	
4	PCLS human (control)	13,500	1,000
4	PCLS human (SARS-CoV-2)	14,500	
**Ki67 positive cells**			
1	NME dog (control)	10,500	0,853
1	NME dog (SARS-CoV-2)	9,000	
2	NME dog (control)	9,000	0,400
2	NME dog (SARS-CoV-2)	20,000	
3	NME dog (control)	6,000	**0,047**
3	NME dog (SARS-CoV-2)	12,000	
4	NME dog (control)	5,500	0,641
4	NME dog (SARS-CoV-2)	7,000	
1	ALI cow (control)	0,355	0,594
1	ALI cow (SARS-CoV-2)	0,500	

5	ALI cow (control)	1,239	**0,004**
5	ALI cow (SARS-CoV-2)	2,786	
1	PCLS dog (control)	3*	0,800
1	PCLS dog (SARS-CoV-2)	3,500	
2	PCLS dog (control)	0*	0,400
2	PCLS dog (SARS-CoV-2)	1,000	
3	PCLS dog (control)	2*	0,800
3	PCLS dog (SARS-CoV-2)	1,000	
4	PCLS dog (control)	5*	0,400
4	PCLS dog (SARS-CoV-2)	0,500	
1	PCLS hamster (control)	2,000	0,400
1	PCLS hamster (SARS-CoV-2)	3,000	
2	PCLS hamster (control)	2,000	0,400
2	PCLS hamster (SARS-CoV-2)	1,000	
3	PCLS hamster (control)	1,000	0,400
3	PCLS hamster (SARS-CoV-2)	2,000	
4	PCLS hamster (control)	2,500	0,607
4	PCLS hamster (SARS-CoV-2)	3,000	
1	PCLS human (control)	6,500	1,000
1	PCLS human (SARS-CoV-2)	5,500	
2	PCLS human (control)	10,500	0,333
2	PCLS human (SARS-CoV-2)	7,000	
3	PCLS human (control)	8,500	1,000
3	PCLS human (SARS-CoV-2)	9,500	
4	PCLS human (control)	3,000	0,667
4	PCLS human (SARS-CoV-2)	4,000	
**CD204 positive cells**			
1	NME dog (control)	8,000	0,631
1	NME dog (SARS-CoV-2)	7,000	
2	NME dog (control)	14,000	0,700
2	NME dog (SARS-CoV-2)	18,000	
3	NME dog (control)	7,000	0,088
3	NME dog (SARS-CoV-2)	12,000	
4	NME dog (control)	4,500	0,115
4	NME dog (SARS-CoV-2)	6,500	
1	PCLS dog (control)	7*	0,800
1	PCLS dog (SARS-CoV-2)	12,000	
2	PCLS dog (control)	2*	0,400
2	PCLS dog (SARS-CoV-2)	8,500	
3	PCLS dog (control)	4*	0,400
3	PCLS dog (SARS-CoV-2)	10,000	
4	PCLS dog (control)	9*	0,800
4	PCLS dog (SARS-CoV-2)	6,500	
1	PCLS hamster (control)	11,000	0,700
1	PCLS hamster (SARS-CoV-2)	8,000	
2	PCLS hamster (control)	7,000	0,100

2	PCLS hamster (SARS-CoV-2)	4,000	
3	PCLS hamster (control)	10,000	0,400
3	PCLS hamster (SARS-CoV-2)	15,000	
4	PCLS hamster (control)	14,500	0,272
4	PCLS hamster (SARS-CoV-2)	19,000	
1	PCLS human (control)	6,000	0,667
1	PCLS human (SARS-CoV-2)	10,000	
2	PCLS human (control)	11,000	1,000
2	PCLS human (SARS-CoV-2)	13,000	
3	PCLS human (control)	8,000	1,000
3	PCLS human (SARS-CoV-2)	10,000	
4	PCLS human (control)	7,500	0,667
4	PCLS human (SARS-CoV-2)	8,500	

[1]* this value is based on one cell culture

[2]The bold font indicates significance.

[3]Abbreviations: ALI, air-liquid interface cultures; dpi, days post infection; n.e., not evaluable, due to lack of target structure; NME, nasal mucosa explants; PCLS, precision-cut lung slices; SARS-CoV-2, severe acute respiratory syndrome coronavirus 2;
